# Antifungal Activity of New Diterpenoid Alkaloids Isolated by Different Chromatographic Methods from *Delphinium peregrinum* L. var. *eriocarpum* Boiss

**DOI:** 10.3390/molecules26051375

**Published:** 2021-03-04

**Authors:** Mohammad Alhilal, Yaser A. M. Sulaiman, Suzan Alhilal, Sobhi M. Gomha, Salama A. Ouf

**Affiliations:** 1 Department of Biochemistry, Faculty of Veterinary Medicine, Ataturk University, 25240 Erzurum, Turkey; 2 Department of Clinical Laboratory Science, College of Pharmacy, Tikrit University, Tikrit 34001, Iraq; neimiyaser@tu.edu.iq; 3 Department of Chemistry, Faculty of Science, University of Albaath, Homs, Syria, 25070 Erzurum, Turkey; salkm13@gmail.com; 4 Chemistry Department, Faculty of Science, Cairo University, Giza 12613, Egypt; 5 Department of Chemistry, Faculty of Science, Islamic University in Almadinah Almonawara, Almadinah Almonawara 42351, Saudi Arabia; 6 Botany & Microbiology Department, Faculty of Science, Cairo University, Giza 12613, Egypt; saoufeg@yahoo.com

**Keywords:** *D. peregrinum eriocarpum* Boiss, diterpenoid alkaloids, Peregrine, antifungal assay

## Abstract

This paper aimed to investigate the potential antifungal influences of new alkaloids from *Delphinium peregrinum* L. var. *eriocarpum* Boiss. New Diterpenoid alkaloids Delcarpum (**1**), Hydrodavisine (**4**) and known alkaloids Peregrine (**2**), Delphitisine (**3**) were isolated by different chromatographic methods from the aerial parts of *D. Peregrinum eriocarpum* Boiss, which grows in Syria. The structures of alkaloids were proposed based on 1D NMR spectroscopy ^1^H-NMR, ^13^C-NMR, DEPT-135, DEPT-90, 2D NMR spectroscopy DQF-COSY, HMQC, EI-Ms mass spectrum, and IR spectroscopic measurements. The antifungal activity of the isolated alkaloids was evaluated against different dermatophyte fungal isolates compared with fluconazole. In the case of Peregrine (**2**) the minimum inhibitory concentrations(MICs) recorded 128–256, 32–64, and 32 for *Epidermophyton floccosum*, *Microsporum canis,* and *Trichophyton rubrum*, respectively, compared to 32–64, 16, and 32 μg/mL in the case of fluconazole, respectively. The MICs recorded on application of the four alkaloids mixture were 64, 32, and 16 in the case of *E. floccosum*, *M. canis*, and *T. rubrum*, respectively, which were significantly lower than that measured for each of the individual alkaloid and were compatible for fluconazole. In conclusion, MICs of the tested alkaloids showed a variable potential effect on the investigated fungal isolates. Peregrine (**2**) was the most effective alkaloid, however, the application of the mixture of alkaloids induced significant synergistic activity that was more pronounced than the application of individual ones.

## 1. Introduction

**Figure 1:**
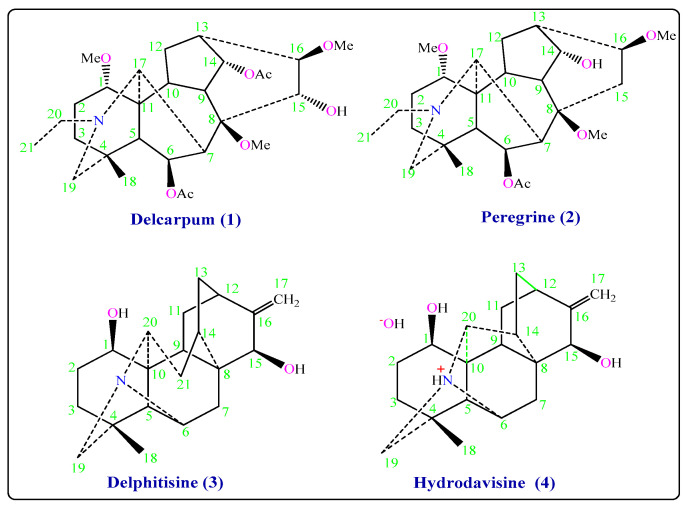
Chemical structures of the isolated compounds (**1**–**4**).

The genus *Delphinium* has been identified in many herbal species in Syria [1]. *Delphinium* is a medical herb used for the treatment of epilepsy, neurotoxic, and asthma [2,3]. The Delphinium alkaloids are currently under investigation in search for new analgesic, antiinflammatory drugs, muscular relaxation, anticonvulsant, pyramis lesion, and cardiac effects [3,4,5]. Moreover, a certain number of natural diterpenoid alkaloids have been reported to possess antiproliferative activities against various human cancer cell lines, indicating their great potential as new drugs for treating the corresponding cancers [6,7]. Due to their diterpenoid alkaloids components, Delphinium species have been considered as insecticidal potential [8]. *Delphinium* species have been used in folk medicine as a parasiticide and for the treatment of itches, skin eruptions. Delphinium was also used in the manufacture of dyes that are used to combat lice [3,9]. The plant *D. peregrinum* L. var. *eriocarpum* Boiss, which grows in Syria, has never been studied before. The first closest plant to it, called *D. peregrinum* var. *elongatum* Boiss, has been collected from Spain. C_19_-diterpenoid alkaloids (bicoloridine, dihydrogadesine, nudicaulidine, 13-acetylhetisinone, peregrine, peregrine alcohol, pergilone, delphiperegrine, and 14-O-acetylperegrine), norditerpenoid alkaloids (dehydrobicoloridine, bicoloridine alcohol, and peregrinine) and C_20_-diterpenoid alkaloids (hetisinone, hetisine, and atisinium chloride) have been isolated from this plant [10,11]. The second closest plant, called *D. peregrinum* has been collected from Turkey. C_19_-diterpenoid alkaloids (peregrine alcohol, pergilone, nudicaulidine, bicoloridine, peregrine, delphiperegrine) have been isolated from this plant [12]. Based on the aforementioned therapeutic effects, *Delphinium* alkaloids are expected to have antifungal effects. According to the available information, the literature has not sufficiently highlighted the expected antifungal effects of the new alkaloids isolated from *D. Peregrinum* L. Var. *Eriocarpum* Boiss in this study. This paper aimed to investigate the potential antifungal influences of new alkaloids isolated by different chromatographic methods from *D. peregrinum* L. var. *eriocarpum* Boiss (Figure 1).

## 2. Results and Discussion

### 2.1. Chemistry

The alkaloids were isolated by column chromatography (C.C) on silica gel of (8.02 g) using a Et_2_O and Et_2_O-CHCl_3_ then CHCl_3_ and CHCl_3_/MeOH/NH_3_ step gradient, followed by further C.C and flash chromatography (F.C), then by preparative TLC. Purification of diterpenoid alkaloids was assigned by TLC, GC, and HPLC.

New alkaloids were determined by mass and NMR data, showed characteristic signals of C_19_-diterpenoid alkaloids and C_20_-diterpenoid alkaloids in their NMR spectra and characteristic fragmentation of such compounds in their mass spectrum [13,14]. The NMR spectra of Delcarpum (**1**). C_28_H_43_NO_8_ gave signals at *δ*_H_ 1.14 (3H, *t*, *J* = 6.99 Hz), *δ*_C_ 11.9 (*q*) of an N-ethyl group, and a signal at *δ*_H_ 0.85 (3H, s), *δ*_C_ 25.2 (*q*) of methyl group, a signal at *δ*_H_ 3.32 and 3.45 (3H, *s*) of two methoxy groups, and one methoxy group at *δ*_H_ 3.17 (3H, *m*). The ^13^C-NMR spectrum (Table 1) of Delcarpum (**1**) gave signals at *δ*_C_ 48.5 (*t*) and *δ*_C_ 25.2 (*q*) of an angular methyl group, and one methoxy group at *δ*_C_ 48.6 (*q*) and at *δ*_C_ 20.2 (*q*), 20.6 (*q*), 170.0 (*s*), and 170.3 (*s*).

The ^13^C-NMR spectrum of Delcarpum (**1**) contained only three signals up field from 81 ppm at *δ*_C_ 32.8 (C-4), 47.6 (C-11), and 80.8 ppm (C-8) indicating the compound possessing is an aconitine-type C-_19_ diterpenoid alkaloids with a tertiary methoxy group at C-8 at *δ*_C_ 48.6 (*q*) [4].

The other two methoxy groups were situated at C-1*α* and C-16*β* to account for the one-proton signals at *δ*_H_ 3.22 (m, H-1*β*) and 3.11 (m, H-16*α*), which, in turn, gave one-bond connectivity with the methine carbon resonant at *δ*_C_ 82.3 (*d*) and 91.4 (*d*) ppm, respectively, in the HMQC spectrum (Table 1). Two acetate groups were situated at C-6*β* and C-14*α* to account for the one-proton signals at *δ*_H_ 5.29 (*d, J* = 7.53, H-6*α*) and 4.67 (*t*, *J* = 4.71 H-14*β*), which are connected with C-6, C-14, which showed a signal *δ*_C_ 71.4 (*d*) and 75.1 (*d*) ppm, respectively, in the HMQC spectrum (Table 1).

**Figure 2:**
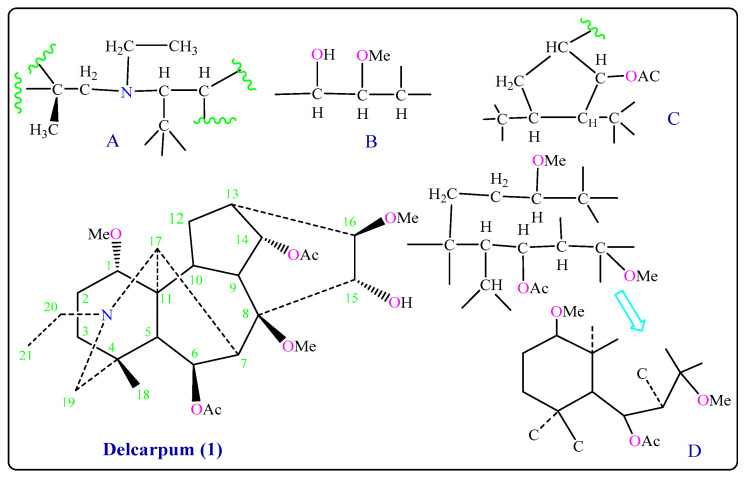
Partial structures of Delcarpum (**1**).

The methine carbon resonances at *δ*_C_ 74.1 (*d*) ppm suggested the presence of the secondary hydroxyl group at *δ*_H_ 3.52 (br *s*, OH) connected with C-15*α* in the molecule [10,11], and one proton of both protons connected with carbon C-12 gave a signal at *δ*_H_ 2.50 (*dd*, *J_1_* = 3.17, *J_2_* = 12.6 Hz, H-12) in which C-12 gave a signal at *δ*_C_ 28.2 (*t*) ppm, while one of the adjacent proton connected with C-13 gave a signal at *δ*_H_ 2.32 (*t*, *J* = 5.54 Hz, H-13) in which C-13 gave a signal at *δ*_C_ 36.4 (*d*) ppm, and two methylene protons showed a signal at *δ*_H_ 2.08 (*d*, *J* = 5.78 Hz, H-19) and *δ*_H_ 2.86 (*d*, *J* = 11.93 Hz, H-19) which are connected with C-19 that gave a signal at *δ*_C_ 56.8 (*t*) ppm. The other ^1^H- and ^13^C-NMR signals (Table 1) were in agreement with the proposed structure, and assignments were made by comparison with spectra of Peregrine (**2**) and ^1^H-COSY and HMQC data (Table 1). After connecting structure parts (A), (B), (C), and (D) we obtained Delcarpum (**1**) (Figure 2 and Figure S1a–g) [15].

The IR spectrum of compound (**1**) (amorphous) (IR *ν*_max_) showed absorbance at 3683–3435 cm^−1^, indicating the presence of OH group and the presence of a strong band at 1725 cm^−1^ indicating carbonyl group (C=O).

The mass spectrum (EIMS) of Delcarpum (**1**) has a peak at *m/z* 521, it represents the molecular ion M^+^ which is corresponding to its molecular weight with formula C_28_H_43_NO_8_, the spectral region adjacent to the M-peak may be used to reveal substituent and functional groups, such as the base peak appears at *m/z* 491, which indicated the presence of methoxy group, which converted into formyl H_2_C=O, CH_3_O (M-31), ethyl (M-29), which are corresponding to the following peaks of *m/z* 490(3), 492(24), respectively. The mass spectrum also gave the fragments of *m/z* -CH_2_OCH_3_ 45, CH_3_-C=O 43

Peregrine (**2**), a colorless crystalline solid, gave a molecular ion at *m/z* 463 (M^+^, 3) in its EIMS, accounting for a molecular formula of C_26_H_40_NO_6_. The chemical structure of compound (**2**) was established according to its IR and NMR spectra (Table 1) [10,11,15].

**Figure 3:**
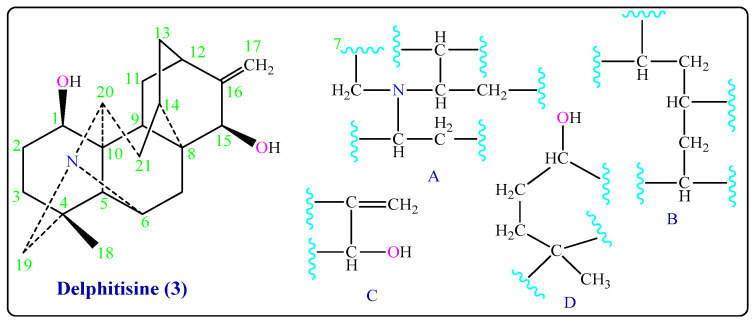
Partial structures of Delphitisine (**3**).

The ^1^H-NMR spectrum of Delphitisine (**3**) exhibited characteristic signals at *δ*_H_ 1.10 (3H, *s*, CH_3_), 1.79 (2H, *m*, 2OH), 4.99 ppm (2H, *d*, C=CH_2_) ppm and tertiary amine with three carbon atoms (60.1, C-19; 2.50 *d*, & 2.69, *d,* H-19), (64.2, C-6; 4.23 *m*, H-6), and (64.4, C-20; 3.56 m, H-20). *The* **^13^***C-NMR spectrum showed twenty-one signals, including one methyl, eight methylene, eight methine, and four quaternary carbons C-4, C-8, C-10, and C-16* that gave signals at *δ*_C_ 35.7, 44.1, 53.4, and 154.1 ppm, respectively (Table 1). The results of COSY and HMQC experiments (in CDCl_3_) and an inspection of literature values reported for C_20_-diterpenoid alkaloids clearly determined the existence of partial structures (A), (B), (C), and (D) (Figure 3 and Figure S2a–g) in Delphitisine (**3**). Its IR spectrum also showed absorption bands at 3633, 3452 (2 -OH), 3056 (=C-H), and 1605 (C=C) cm^−1^. Moreover, the mass spectrum (EIMS) of Delphitisine (**3**) has a peak at *m/z* 327 (54%) represents the molecular ion M^+^, which is corresponding to its molecular weight and a molecular formula of C_21_H_29_NO_2_. All that data can be satisfactorily supported by the structure of Delphitisine (**3**). The closest type is Hetisine-type, which is one of the most complex entries in the atisane-class [10,11,16,17].

**Figure 4:**
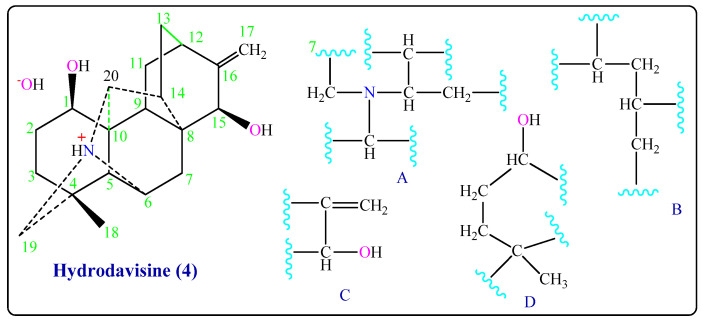
Partial structures of Hydrodavisine (**4**).

The study spectrums of Hydrodavisine (**4**) gave signals at *δ*_H_ 5.01ppm (2H, *d*, *J* = 7.21 Hz), of an (-C=CH_2_), signals at *δ*_C_ 108.1, 154.2 ppm of quaternary carbon sp^2^, and IR in cm^−1^ at 1620 (C=C), 3019 (=C-H). The signal at *δ*_H_ (2H, 4, 89 (*m*) ppm) of an (-OH) group and in IR (3683–3620 & 3460) cm^−1^, with hydrogen bonding. A methyl group -CH_3_ was founded by *δ*_H_ 1.16 (3H, *s*) ppm and its *δ*_C_ 26.6 ppm (Table 1). The group (C_3_N^+^H) has been shown by signals at *δ*_C_ (C_6_: 63.6, C_19_: 59.4, C_20_: 66.3 ppm) and a hydrogen atom (1H, 3.74 ppm). **^13^***C-NMR spectrum showed twenty signals, including one methyl, seven methylene, eight methine, and four quaternary carbons C-4, C-8, C-10, and C-16* that gave signals at *δ*_C_ 35.4, 36.3, 54.2, and 154.2 ppm, respectively (Table 1). After studied COSY and HMQC experiments (in CD_3_OD) it found the partial structures (A), (B), (C), and (D) (Figure 4 and Figure S3a–h), and after comparing the ^13^C-NMR of Hydrodavisine (**4**) with its of Delphitisine (**3**) and of C_20_-diterpenoid alkaloids [5,10,11]. We made sure that this compound has the same skeleton of Delphitisine (**3**), losing a secondary carbon atom. In addition to that, the mass spectrum of Hydrodavisine (**4**) (EIMS) the molecular weight M^+^ = 331 is corresponding to its molecular formula of [C_20_H_29_NO_3_], a peak at *m/z* 314 (96%) represents the molecular ion (M-OH)^+^. The peak at *m*/*z* 348 was for the fragment [M + OH]^+^, and the peak *m*/*z* 349 was for the fragment (M + H_2_O)^+^. The peak in ^1^H-NMR 4.89 ppm for OH hydrogen bonds with water molecules [15,18].

### 2.2. Biology

#### Antifungal Activity

MIC of the tested alkaloids showed a different potential effect on the investigated fungal isolates. Peregrine was the most effective where the MIC recorded 128–256, 32–64, and 32 for *Epidermophyton floccosum*, *Microsporum canis*, and *Trichophyton rubrum*, respectively, compared to 32–64, 16, and 32 μg/mL in the case of fluconazole. Alkaloids extracted from the plant have been known to have important characteristics with biochemical, pharmacological, and medical effects in living organisms [19,20]. The investigators isolated three C_19_-diterpenoid alkaloids: Delbrunine, delbruline, and delbrusine, from *Delphinium brunonianum* and they indicated that these compounds have an antibacterial effect against *Escherchia coli*, *Staphylococcus aureus*, *Pseudomonas aureginous*, *Salmonella flexinarie,* and *Bacillus subtilis*. Sometimes, the use of individual bioactive compounds extracted from a particular plant does not induce the predicted inhibitory effects compared to its original synergistic combination with other associate candidates. The MIC and MFC of the mixture of tested alkaloids were significantly more pronounced than the application of individual ones. The MICs recorded on the application of the mixture of the four alkaloids were 64, 32, and 16 in the case of *E. floccosum, M. canis,* and *T. rubrum*, respectively, which was significantly lower than that measured for each of the individual alkaloids and were compatible for fluconazole as reference standard drug (Table 2). Hemaiswarya et al. [21] indicated that several plant extracts have shown synergistic activity against microorganisms. This review designates in detail the observed synergy and mechanism of action between natural products including flavonoids and essential oils and synthetic drugs in combating microbial infections. The mode of action of combination differs significantly from that of the same drugs acting individually, hence, isolating a single component may lose its therapeutic importance.

## 3. Materials and Methods

### 3.1. Chemistry

*General*. IR: CHCl_3_ for compounds (**1**, **2**, and **3**) and MeOH for compound (**4**) on FT-IR spectrum (Impact 410-Nicolet Madison, WI, USA). MS spectra were recorded on Varian GC/MS EI MS 70 eV. 3800 gas chromatograph directly interfaced with 2000 ion trap mass spectrometer (Varian, Walnut Creek, CA, USA). NMR spectra were recorded in CDCl_3_ for compounds (**1**, **2**, and **3**) and CD_3_OD for compound (**4**) on Bruker Avance 300 MHz (Bruker Biospin, Karlsruhe, Germany). Silica gel pH = 7, 230 mesh was used for column chromatography (C.C), pH = 7, 400 mesh was utilized for thin-layer chromatography (TLC) and silica gel 60 F254, TLC plates (Merck, Darmstadt Germany). All chemicals used in this study were obtained from Merck (Merck, Darmstadt, Germany).

*Plant material:* The investigated plant is related to the family Ranunculaceae, annual with herbaceous growth of 10–60 cm tall. The aerial parts (1 kg) were collected from several localities located at Umm Harten village between Homs and Tartous cities, Syria, during the flowering period (June to August) and authenticated in Botany Department, Faculty of Science, University of Damascus.

*Extraction and isolation*: Air-dried parts were extracted with methanolic NH_3_ (2%) at room temperature for a week. The resulting extract (208.5 g) was treated with 5% H_2_SO_4_. The acid was neutralized with 25% NaOH and extracted with Et_2_O then with CHCl_3_ to give crude alkaloid material (9.29 g). Column chromatography (C.C) on silica gel (8.02 g) using Et_2_O and Et_2_O/CHCl_3_ then CHCl_3_ and CHCl_3_/MeOH/NH_3_ step gradient, followed by further C.C flash chromatography (F.C) and preparative TLC.

Delcarpum (**1**): 67 mg resin were isolated by preparative TLC (Et_2_O:Me_2_CO 5:1), Rf = 0.212 in (MeOH:CH_2_Cl_2_ 1:1), and TLC (C_6_H_6_:CH_2_Cl_2_:MeOH 1:6:3) R_f_ = 0.8, respectively. Peregrine (**2**): 56 mg crystalline, mp 123.5–125 °C from (hexane:EtOAc 1:1). Delphitisine (**3**): 31 mg resin was isolated by preparative TLC (MeOH:CH_2_Cl_2_ 1:1) R_f_ = 0.515 and Hydrodavisine (**4**): 27 mg white crystals was isolated by preparative TLC (MeOH:CH_2_Cl_2_ 1:1) recrystallization (CH_2_Cl_2_:MeOH 90:10) mp (dec) = 304–309 °C; R_f_ = 0.25 in (MeOH:CH_2_Cl_2_ 1:1).

Purification of Delcarpum (**1**), Peregrine (**2**), Delphitisine (**3**) and Hydrodavisine (**4**) was assigned by TLC, GC, and HPLC. The structures were identified by 1D NMR spectroscopy ^1^H-NMR, ^13^C-NMR, DEPT-135, DEPT-90, 2D NMR spectroscopy DQF-COSY, HMQC, EI-Ms mass spectrum, and IR spectroscopy (Figure 1).

Delcarpum (**1**): Amorphous IR νmax CHCl_3_ cm^−1^: 3683, 3435, 3009, 2946, 1725, 1210, 1082, 784; ^1^H-NMR (300 MHz, CDCl_3_): *δ* 0.85 (3H, s), 1.14 (3H, *t*, *J* = 6.99 Hz), 2.08 (1H, *d*, *J* = 5.78 Hz), 2.32 (H, *t*, *J* = 5.54 Hz), 2.50 (H, *dd*, *J_1_* = 3.17, *J_2_* = 12.6 Hz), 2.86 (1H, *d*, *J* =11.93 Hz), 3.32 and 3.45 (3H each, *s*, 2 × OMe), 4.18 (H, *d*, *J* = 6.91 Hz), 4.67(H, *t*, *J* = 4.71 Hz), 3.52 (1H, br *s*, OH), 5.29 (H, *d*, *J* = 7.53 Hz); EIMS *m*/*z* (rel.int.) 521 (M^+^, 1), 492 (24), 491 (100), 490 (3), 431 (3), 401 (3), 400 (3), 98 (5), 91 (6), 79 (5), 77 (5), 58 (24), 56 (5), 45 (6), 44 (5), 43 (92), 42 (6), 41 (5). For ^13^C-NMR (300 MHz), see Table 1 and Table S1–S4.

Peregrine (**2**): Crystalline solid, mp = 123.5–125 °C from hexane-EtOAc; [α]D + 20° 9c 0.2). IR νmax KBr cm^−1^: 3633, 3438, 3001, 2946, 1733, 1230, 1081, 796; ^1^H-NMR (300 MHz, CDCl_3_): *δ* 0.85 (3H, *s*), 1.07 (3H, *t*, *J* = 7.12 Hz), 1.25 (1H,), 1.48 (1H, *s*), 1.57 (1H, -), 1.88 (1H, *m*), 2.01 (2H, -), 2.08 (7H, -), 2.25 (1H, *dd*, *J* = 5.31 Hz), 2.35 (1H, *t*, *J* = 5.67 Hz), 2.47 (2H, *m*), 2.61 (1H, *d*, *J* = 11.90 Hz), 2.74 (1H, *d*, *J* = 7.29 Hz), 3.05 (1H, *t*, *J* = 5.53 Hz), 3.11 (4H, -), 3.16 (1H, *d*, *J* = 2.07 Hz), 3.28 (3H, *s*), 3.39 (4H, -), 3.70 (1H, *d*, *J* = 6.48 Hz, OH), 4.02 (1H, *q*, *J* = 10.585 Hz), 5.25 (1H, *d*, *J* = 7.28 Hz); EIMS *m*/*z* (rel.int.) 463 (M^+^, 3), 434 (16), 433 (77), 432 (17), 403 (9), 402 (13), 401 (23), 400 (13), 373 (27), 372 (21), 341 (17), 340 (15), 123 (12), 96 (10), 91 (20), 79 (12), 77 (15), 71 (17), 65 (10), 58 (38), 56 (11), 55 (12), 45 (13), 43 (100), 42 (11), 41 (18). For ^13^C-NMR (300 MHz) see Table 1 and Table S1–S4. Peregrine (**2**) has been isolated before) [10,11,12].

Delphitisine (**3**): Amorphous IR νmax CHCl_3_ cm^−1^: 3633, 3438, 1605; ^1^H-NMR (300 MHz, CDCl_3_): *δ* 1.08 (3H, *s*), 1.10 (1H, *m*), 1.29 (3H, *m*), 1.77 (2H, *m*),1.79(6H, *m*) 1.94 (2H, *m*), 1.99 (1H, *m*), 2.07 (3H, *m*), 2.26 (1H, *d*, *J* = 2.29 Hz), 2.50 (1H, *d*, *J* = 12.45 Hz), 2.67 (1H, *s*), 2.69 (1H, *d*, *J* = 12.44 Hz), 3.56 (1H, *m*), 4.05 (1H, *s*), 4.23 (1H, *m*), 4.99 (1H, *d*, *J* = 7.60 Hz); EIMS *m*/*z* (rel.int.) 328 (M^+^ + 1, 13), 327 (M^+^, 54), 326 (100), 325 (13), 311 (20), 309 (75), 308 (32), 307 (14), 167 (19), 150 (24), 134 (20), 132 (24), 131 (24), 120 (32), 118 (41), 110 (22), 109 (25), 107 (38), 105 (21), 95 (29), 94 (30), 93 (21), 92 (81), 82 (25), 81 (26), 80 (61), 79 (27), 78 (73), 77 (16), 70 (19), 67 (38), 65 (10), 58 (38), 55 (43), 53 (38), 51 (38), 50 (12), 44 (32), 43 (35), 42 (26), 41 (71), 40 (15). For ^13^C-NMR (300 MHz), see Table 1 and Table S1–S4.

Hydrodavisine (**4**): Crystalline solid, mp(dec) = 304–309 °C from (MeOH:CH_2_Cl_2_); IR νmax MeOH cm^−1^: 3683, 3620, 3460, 3019, 2975, 2400, 1620,1521, 1423, 1213, 1046, 929, 759, 669; ^1^H-NMR (300 MHz, CD_3_OD): *δ* 1.16 (3H, *s*), 1.38 (3H, *m*), 1.70 (2H, *m*), 1.84 (4H, *m*), 1.90 (1H, *m*), 2.11 (2H, *m*), 2.21 (1H, *m*), 2.25 (1H, *m*), 2.86, (2H, *q*, *J* = 12.29 Hz), 3.10 (1H, *s*), 3.74 (^+^NH, *m*), 3.93 (1H, *s*), 3.99 (1H, *s*), 4.20 (1H, *s*), 4.89 (2OH + 2H_2_O, br *s*), 5.01 (2H, *d*, *J* = 7.21 Hz); EIMS *m*/*z* (rel.int.) 349(M^+^ + 18, 3), 348(M^+^ + 17, 5), 331 (M^+^, 3), 315 (M^+^ − O, 11), 314 (M^+^ − OH, 96), 313 (24), 312 (7), 311 (4), 299 (11), 298 (36), 297 (100), 296 (82), 295 (36), 294 (26), 293 (21), 292 (14), 291 (10), 277 (10), 276 (10), 252(8), 158 (10), 157 (11), 154 (10), 143 (10), 120 (13), 110 (10), 109 (12), 108 (12), 107 (11), 106 (11), 56 (7), 44 (10), 43 (7), 42 (12), 40 (6); In another MS: 331 (M^+^): [C_20_H_28_^+^NO_2_ + ^−^OH], 349 (M +1 8): [C_20_H_28_^+^NO_2_ + ^−^OH + H_2_O]. For ^13^C-NMR (300 MHz), see Table 1 and Table S1–S4.

### 3.2. Biology

Test organisms: Five different dermatophyte isolates from *Epidermophyton floccosum*, *Microsporum canis*, and *Trichophyton rubrum* were tested in this investigation. The test isolates were chosen from the identified stock specimens of the author and were isolated from patients with superficial clinical infections with different tinea infections admitted to dermatological and microbiological laboratories in Cairo, Egypt [22]. The inoculums were prepared from 10-day-old cultures. The isolates were maintained in Sabouraud dextrose agar (SDA) (Oxoid, Basingstoke, UK) with 20% glycerol, preserved at −80 °C, and cultured on potato dextrose agar (PDA) (Oxoid) plates incubated at 30 °C before investigation.

Antifungal activity: Assay of minimum inhibitory concentration (MIC): The experimental design was achieved according to CLSI M38-A protocols for filamentous fungi using the broth microdilution method following the Clinical and Laboratory Standards Institute (CLSI) M38-A2 guidelines [23]. Briefly, a fungal suspension was scraped from the margin of actively growing mycelium grown at 30 °C on potato dextrose agar (PDA). One percent solution Tween 80 in distilled water was added to the scraped mycelium, and the suspension was diluted 1:50 (*v*/*v*) in RPMI-1640 culture medium (Sigma, Darmstadt, Germany) in presence of a buffer, where the suspension contained about 5.0 × 10^4^ CFU/mL. The compounds were prepared in RPMI medium supplemented with MOPS. Serial dilutions 1:2 were performed on the microplates and evaluated at bifold concentrations from 1 to 1024 μg/mL of each of the tested compounds or their mixture. Untreated controls were then tested, and 100 μL of the initially prepared inoculum was added to the microplates. The antifungal effect was observed after seven days of incubation at 30 °C by optical observation of turbidity. MIC was the lowest concentration of compound capable of inhibiting observed fungal growth in the wells by 100%. Fluconazole was used as an antifungal reference drug. All experiments were performed in triplicate.

Minimum fungicidal concentration (MFC): After recording the MIC value for each sample, 10 μL from clear wells, the last tube showing growth, were subcultured on Sabouraud dextrose agar medium (OXOID LTD, CM0041). The dishes were incubated at 30 °C for 7 days. A control without drugs was performed. MFC was defined as the lowest concentration of the compound at which growth was <3 CFU. All experiments were performed in triplicate.

## 4. Conclusions

Four alkaloids were isolated from the aerial parts of *D. Peregrinum eriocarpum* Boiss, two of them are from C_19_-diterpenoid alkaloids: Delcarpum (**1**) and Peregrine (**2**). The other two alkaloids are from C_20_-diterpenoid alkaloids: Delphitisine (**3**) and Hydrodavisine (**4**). Delcarpum (**1**) and Hydrodavisine (**4**) alkaloids are isolated for the first time. Concerning the antifungal effect, Peregrine (**2**) is the most effective in comparison with other alkaloids. In addition, the mixture of tested alkaloids is significantly inhibitory and is more pronounced than the application of individual ones.

## Figures and Tables

**Table 1:** ^13^C-NMR & HMQC NMR data assignments for Delcarpum (**1**), Peregrine (**2**), Delphitisine (**3**), and Hydrodavisine (**4**).

Position	Delcarpum 1 CDCl_3_		Peregrine 2 CDCl_3_		Delphitisine 3 CDCl_3_		Hydrodavisine 4 MeOD	
		HMQC *δ*_H_		HMQC *δ*_H_		HMQC *δ*_H_		HMQC *δ*_H_
	*δ*_C_	(*mult.*, *J* = Hz)	*δ*_C_	(*mult*., *J* = Hz)	*δ*_C_	(*mult.*, *J* = Hz)	*δ*_C_	(*mult.*, *J* = Hz)
1	82.3	3.22 *m*	84.1	3.11 -	73.3	2.67 *s*	72.9	3.10 *s*
2	24.6	1.87 *m*	25.9	2.01 -	25.1	1.79 *m*	31.5	1.38 *m*
		2.15 -		2.08 -		1.94 *m*		
3	28.8	1.25 *m*	36.5	1.25 -	28.1	1.29 *m*	25.9	1.84 *m*
				1.57 -				1.90 *m*
4	32.8	-	33.9	-	35.7	-	35.4	-
5	54	1.50 *s*	55.7	1.48 *s*	54.1	1.99 *m*	54	2.21 *m*
6	71.4	5.29 *d (7.53*)	72.8	5.25 *d (7.28)*	64.2	4.23 *m*	63.6	4.20 *s*
7	35.2	3.17 -	41.7	2.75 *d (7.29)*	26	1.29 *m*	25.9	1.84 *m*
						1.79 *m*		
8	80.8	-	78.5	-	44.1	-	36.3	-
9	40.6	3.35 -	44	3.05 -	31.8	2.26 *d (2.29)*	33.1	2.25 *m*
10	44.3	2.15 -	45.6	2.01 -	53.4	-	54.2	-
11	47.6	-	47.6	-	31.3	1.10 *m*	30.9	1.70 *m*
						1.79 *m*		
12	28.2	1.97 -	28	1.88 *m*	39.8	2.07 *m*	42.3	2.11 *m*
		2.50 *dd (3.17*, *12.6)*		2.25 *dd* (5.31)				
13	36.4	2.32 *t (5.54)*	37.9	2.35 *t (5.67)*	25.4	1.79 *m*	26.6	1.38 *m*
						1.94 *m*		1.84 *m*
14	75.1	4.67 *t (4.71)*	74.8	4.02 *q (10.59)*	41.1	2.07 *m*	41.4	2.11 *m*
15	74.1	4.18 *d (6.91)*	32.5	2.08 -	69.4	4.05 *s*	69.5	3.99 *s*
16	91.4	3.11 -	81.9	3.39 -	154.1	-	154.2	-
17	62.8	3.22 -	64.3	3.16 *d (2.07)*	107.6	4.99 *d (7.60)*	108.1	5.01 *d (7.21)*
18	25.2	0.85 s	25.3	0.85 *s*	26.7	1.08 *s*	26.6	1.16 *s*
19	56.8	2.08 *d (5.78)*	56. 9		60.1	2.50 *d (12.45)*	59.4	2. 86 *q (12.29)*
		2.86 *d (11.93)*				2.69 *d (12.44)*		
20	48.5	2.63 *m*	48.7	2.47 *m*	64.4	3.56 *m*	66.3	3.93 s
		2.72 *m*						
21	11.9	1.14 *t (6.99)*	13.1	1.07 *t (7.12)*	30.4	1.79 *m*	-	-
						2.07 *m*		
1′	55	3.32 *s*	55. 6	3.28 *s*	-	-	-	-
6′	170.3	-	170.7		-	-	-	-
6′′	20.6	1.97 -	21.2	2.08 -	-	-	-	-
8′	48.6	3.17 -	47.7	3.11 -	-	-	-	-
14′	170	-	-	-	-	-	-	-
14′′	20.2	1.97 -	-	-	-	-	-	-
16′	56.2	3.45 s	55.9	3.39 -	-	-	-	-

Chemical shifts in *δ* (ppm) down-field from TMS coupling constants *J* in Hz (*s*: singlet, *d*: doublet, *t*: triplet, *q*: quartet, *m*: multiplet). Carbon multiplicities determined by DEPT-135.

**Table 2:** Range of minimum inhibitory (MIC) and minimum fungicidal concentrations (MFC) of synthesized alkaloid isolated from *Delphinium peregrinum* L. var. *eriocarpum* Boiss against different dermatophyte fungal isolates measured as µg/mL.

Alkaloid Derivative	Dermatophytes (Five Isolates Tested for Each Species)					
	*Epidermophyton floccosum*		*Microsporum canis*		*Trichophyton rubrum*	
	MIC	MFC	MIC	MFC	MIC	MFC
Delcarpum (**1**)	265	512	64	128–265	32–64	256
Peregrine (**2**)	128–256	>512	32–64	128	32	128
Delphitisine (**3**)	512	>512	128–265	512	64–128	512
Hydrodavisine (**4**)	512	>512	128	512	64–128	512
Mixture of the four alkaloids	64	128	32	128–258	16	64
Fluconazole	32–64	64	16	32–64	32	64

The mean value of replicates for MIC and MFC was calculated after considering the standard deviation for the data.

## Data Availability

The data presented in this study are available in this article.

## Supplementary Materials

The following are available online, Figure S1a–g. Spectral characterization of Delcarpum (**1**), Figure S2a–g. Spectral characterization of Delphitisine (**3**), Figure S3a–h. Spectral characterization of Hydrodavisine (**4**), Table S1. ^1^H, ^1^H COSY (Correlation Spectroscopy Homonuclear) and HMQC (Heteronuclear Multiple Quantum Coherence) NMR data for Delcarpum (**1**), Table S2. ^1^H, ^1^H COSY (Correlation Spectroscopy Homonuclear) and HMQC (Heteronuclear Multiple Quantum Coherence) NMR data for Peregrine (**2**), Table S3. ^1^H, ^1^H *COSY* (Correlation Spectroscopy Homonuclear) and HMQC (Heteronuclear Multiple Quantum Coherence) NMR data. for Delphitisine (**3**) and Table S4. ^1^H, ^1^H COSY (Correlation Spectroscopy Homonuclear) and HMQC (Heteronuclear Multiple Quantum Coherence) NMR data for Hydrodavisine (**4**).

## Sample Availability

Samples of the compounds are not available from the authors.
