# Supplementary material for: Antifungal Activity of New Diterpenoid Alkaloids Isolated by Different Chromatographic Methods from Delphinium peregrinum L. var. eriocarpum Boiss
Source: Molecules. 2021 Mar 4;26(5):1375. doi: 10.3390/molecules26051375 (PMC7961722; doi:10.3390/molecules26051375)
Supplement: Supplementary file 1 [file molecules-26-01375-s001.pdf]

Antifungal Activity of New Diterpenoid Alkaloids Isolated by Different Chromatographic Methods from *Delphinium Peregrinum L. Var. Eriocarpum Boiss*

Mohammad Alhilal [1,*], Yaser A.M. Sulaiman [2], Suzan Alhilal [3], Sobhi M. Gomha [4,5,*] and Salama A. Ouf [6]

[1] Department of Biochemistry, Faculty of Veterinary Medicine, Ataturk University, Erzurum 25240, Turkey
[2] Department of Clinical Laboratory Science, College of Pharmacy, Tikrit University, Tikrit 34001, Iraq; neimiyaser@tu.edu.iq
[3] Department of Chemistry, Faculty of Science, University of Albaath, Homs, Syria, Erzurum 25070, Turkey; salkm13@gmail.com
[4] Chemistry Department, Faculty of Science, Cairo University, Giza 12613, Egypt
[5] Department of Chemistry, Faculty of Science, Islamic University in Almadinah Almonawara, Almadinah Almonawara 42351, Saudi Arabia
[6] Botany & Microbiology Department, Faculty of Science, Cairo University, Giza 12613, Egypt; saoufeg@yahoo.com
* Correspondence: mohammad.alhilal15@ogr.atauni.edu.tr or dr.mkh.1981@gmail.com (M.A.); sm.gomha@iu.edu.sa or s.m.gomha@gmail.com (S.M.G.)

<u>**Table of Contents:**</u>

Fig. S1a. The IR spectrum of Delcarpum (**1**) in $CHCl_3$.
Fig. S1b. The EIMS spectrum of Delcarpum (**1**).
Fig. S1c. The ^{1}H-NMR spectrum of Delcarpum (**1**) in $CDCl_3$ Bruker 300 MHz.
Fig. S1d. The ^{13}C-NMR spectrum of Delcarpum (**1**) in $CDCl_3$ Bruker 300 MHz.
Fig. S1e. The DEPT-135 expand spectrum of Delcarpum (**1**) in $CDCl_3$ Bruker 300 MHz.
Fig. S1f. The ^{1}H/^{13}C HMQC spectrum of Delcarpum (**1**) in $CDCl_3$ Bruker 300 MHz.
Fig. S1g. The ^{1}H/^{1}H DQF-COSY spectrum of Delcarpum (**1**) in $CDCl_3$ Bruker 300 MHz.
Fig. S1a-g. Spectral characterization of Delcarpum (**1**)
Fig. S2a. The IR spectrum of Delphitisine (**3**) in $CHCl_3$.
Fig. S2b. The EIMS spectrum of Delphitisine (**3**).
Fig. S2c. The ^{1}H-NMR spectrum of Delphitisine (**3**) in $CDCl_3$ Bruker 300 MHz.
Fig. S2d. The ^{13}C-NMR spectrum of Delphitisine (**3**) in $CDCl_3$ Bruker 300 MHz.
Fig. S2e. The DEPT-135 spectrum of Delphitisine (**3**) in $CDCl_3$ Bruker 300 MHz.
Fig. S2f. The ^{1}H/^{13}C HMQC spectrum of Delphitisine (**3**) in $CDCl_3$ Bruker 300 MHz.
Fig. S2g. The ^{1}H/^{1}H DQF-COSY spectrum of Delphitisine (**3**) in $CDCl_3$ Bruker 300 MHz.
Fig. S2a-g. Spectral characterization of Delphitisine (**3**).
Fig. S3a The IR spectrum of Hydrodavisine (**4**) in MeOH.
Fig. S3b. The EIMS spectrum of Hydrodavisine (**4**)
Fig. S3c. The ^{1}H-NMR spectrum of Hydrodavisine (**4**)in CD_3OD Bruker 300 MHz.
Fig. S3d. The ^{13}C-NMR spectrum of Hydrodavisine (**4**)in CD_3OD Bruker 300 MHz.
Fig. S3e. The DEPT-135 spectrum of Hydrodavisine (**4**)in CD_3OD Bruker 300 MHz.
Fig. S3f. The DEPT-90 spectrum of Hydrodavisine (**4**) in CD_3OD Bruker 300 MHz.
Fig. S3g. The ^{1}H/^{13}C HMQC spectrum of Hydrodavisine (**4**)in CD_3OD Bruker 300 MHz.
Fig. S3h. The ^{1}H/^{1}H COSY spectrum of Hydrodavisine (**4**) in CD_3OD Bruker 300 MHz.
Fig. S3a-h. Spectral characterization of Hydrodavisine (**4**).
Table S1.^{1}H, ^{1}H COSY(Correlation Spectroscopy Homonuclear) and HMQC(Heteronuclear Multiple Quantum Coherence) NMR data for Delcarpum (**1**).
Table S2. ^{1}H, ^{1}HCOSY(Correlation Spectroscopy Homonuclear) and HMQC (Heteronuclear Multiple Quantum Coherence) NMR data for Peregrine (**2**).
Table S3. ^{1}H, ^{1}HCOSY*(Correlation Spectroscopy Homonuclear) and HMQC (Heteronuclear Multiple Quantum Coherence)* NMR data. for Delphitisine **(3).**
Table S4. ^{1}H, ^{1}HCOSY *(Correlation Spectroscopy Homonuclear) and HMQC (Heteronuclear Multiple Quantum Coherence)* NMR data for *Hydrodavisine* **4.**

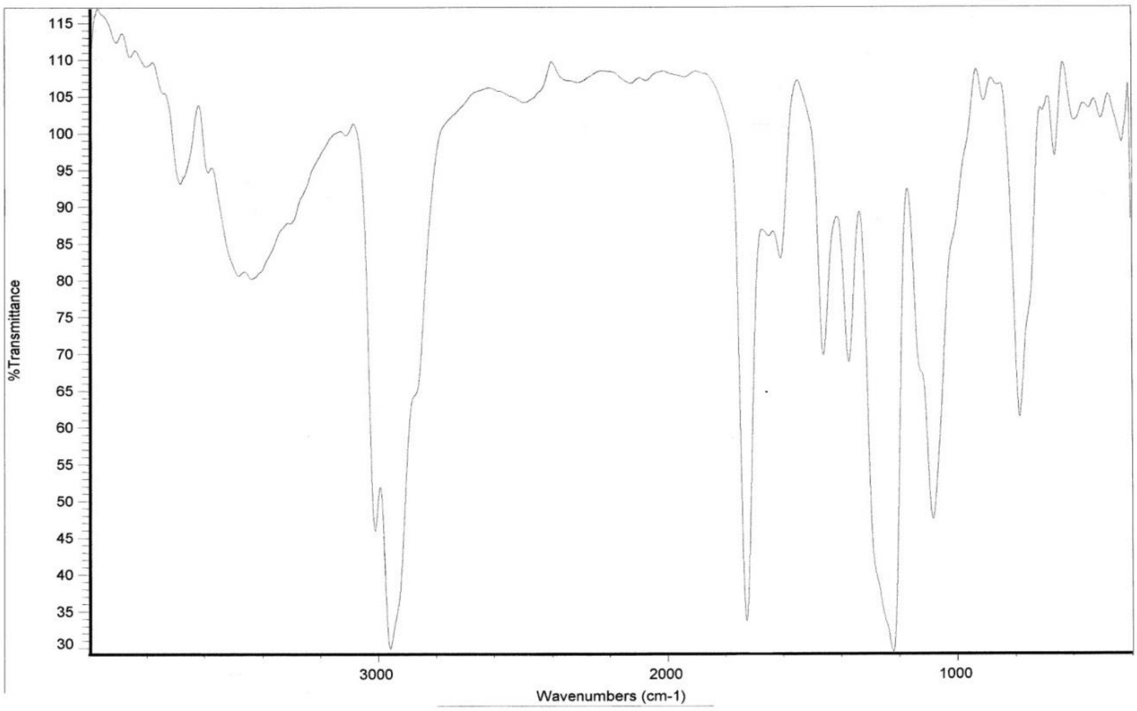

Fig. S1a. The IR spectrum of Delcarpum (**1**) in $CHCl_3$.

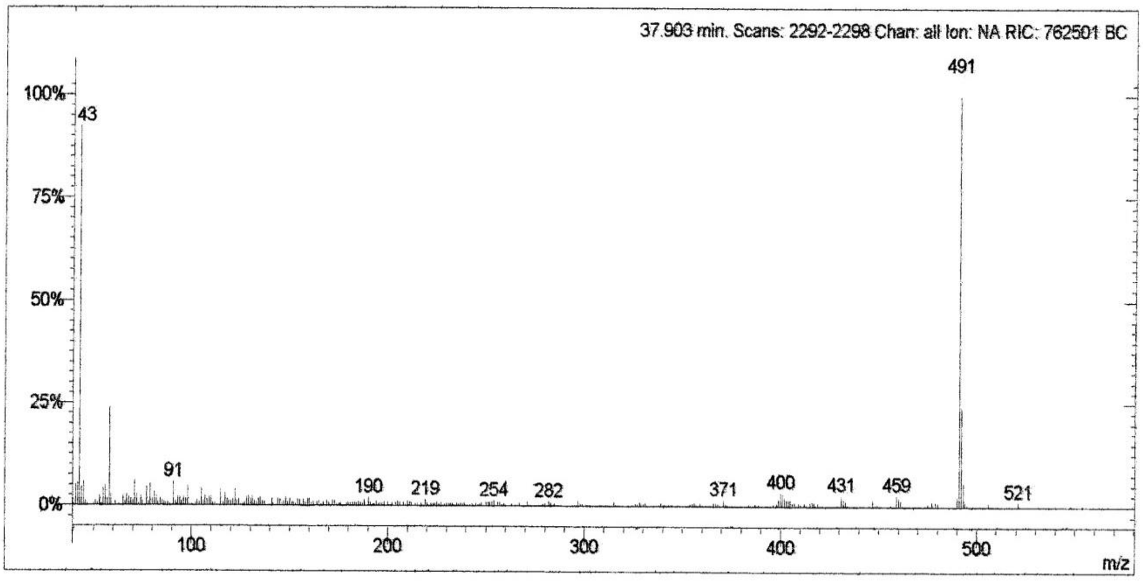

Fig. S1b. The EIMS spectrum of Delcarpum (**1**).

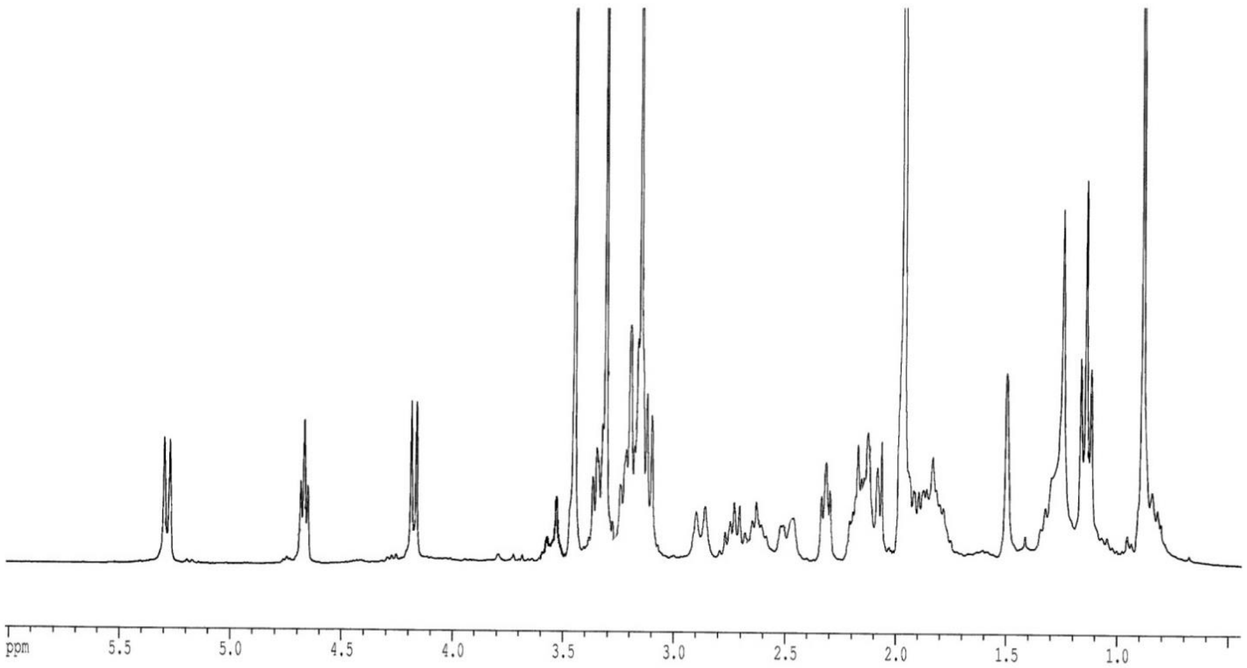

Fig. S1c. The ^{1}H-NMR spectrum of Delcarpum (**1**) in $CDCl_3$ Bruker 300 MHz.

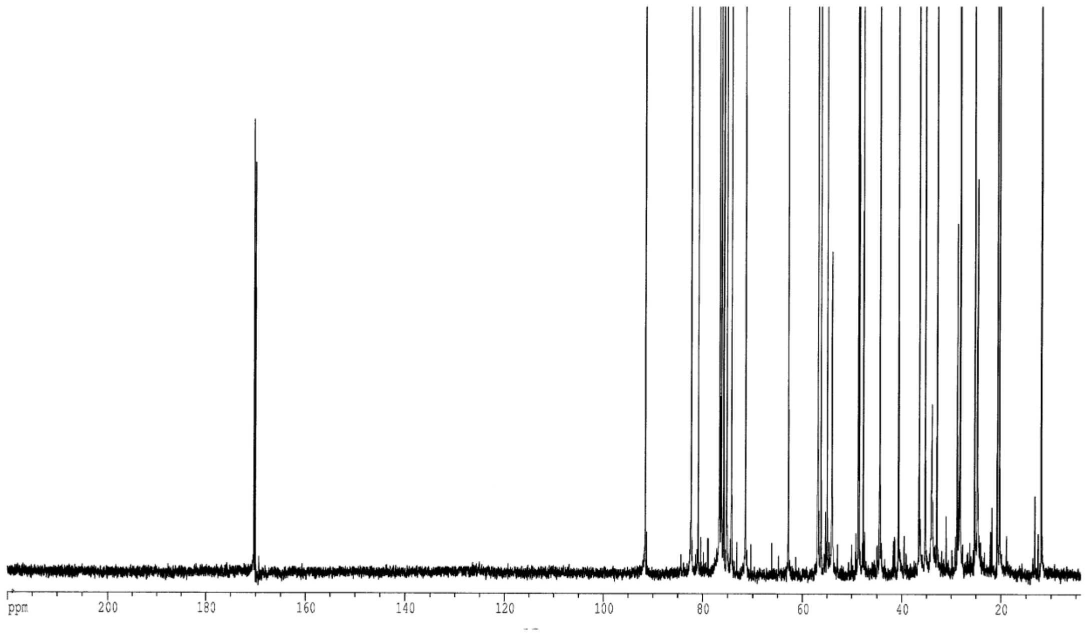

Fig. S1d. The ^{13}C-NMR spectrum of Delcarpum (**1**) in $CDCl_3$ Bruker 300 MHz.

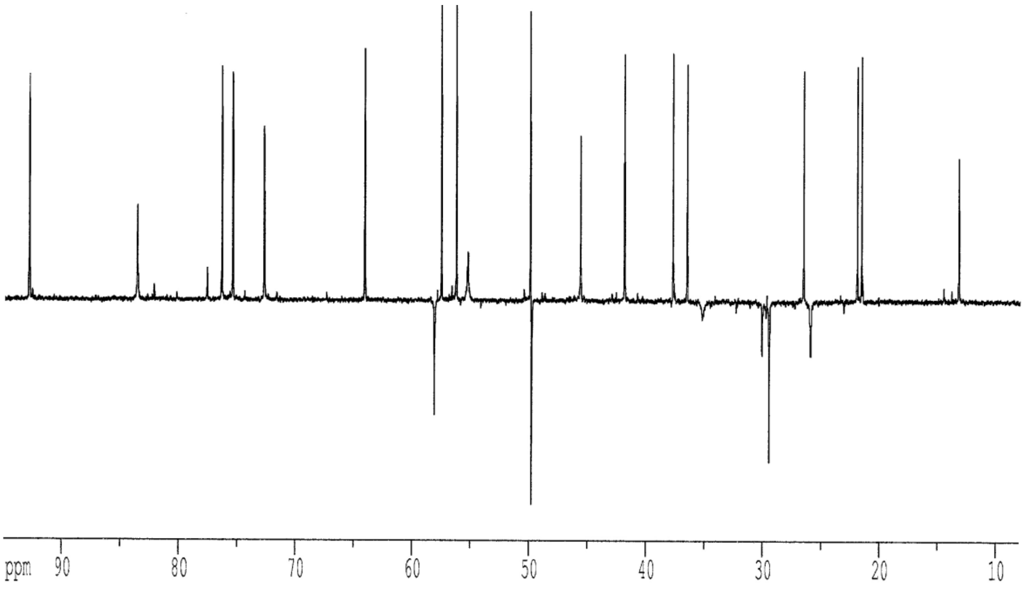

Fig. S1e. The DEPT-135 expand spectrum of Delcarpum (**1**) in $CDCl_3$ Bruker 300 MHz.

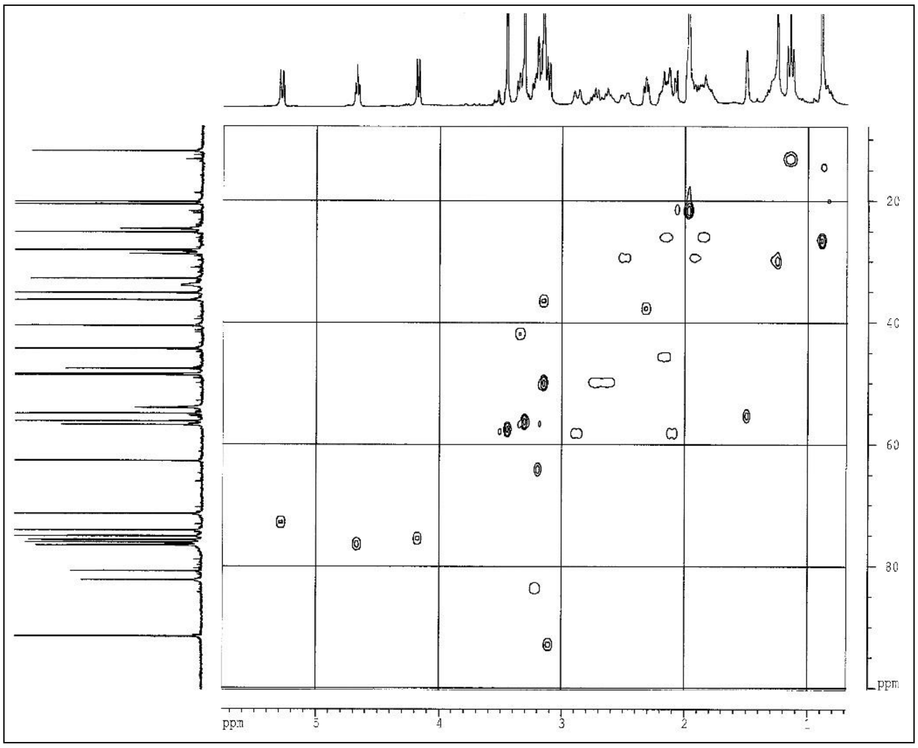

Fig. S1f. The $^{13}C/^{1}H$ HMQC spectrum of Delcarpum (**1**) in $CDCl_3$ Bruker 300 MHz.

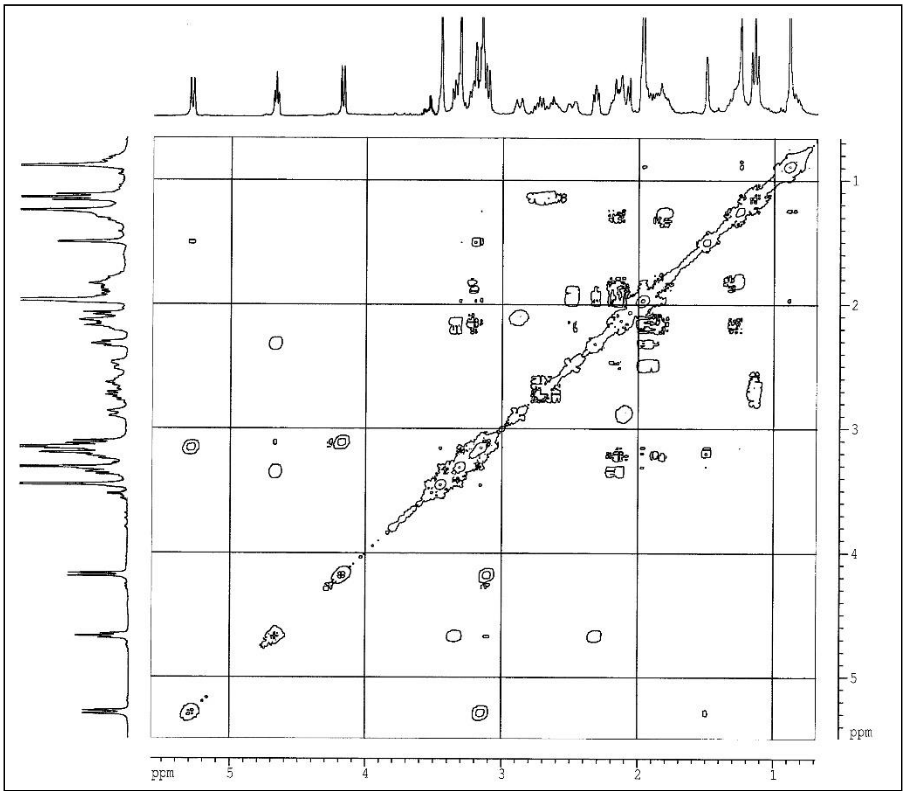

Fig. S1g. The $^1H/^1H$ DQF-COSY spectrum of Delcarpum (**1**) in $CDCl_3$ Bruker 300 MHz.

Fig. S1a-g. Spectral characterization of Delcarpum (**1**)

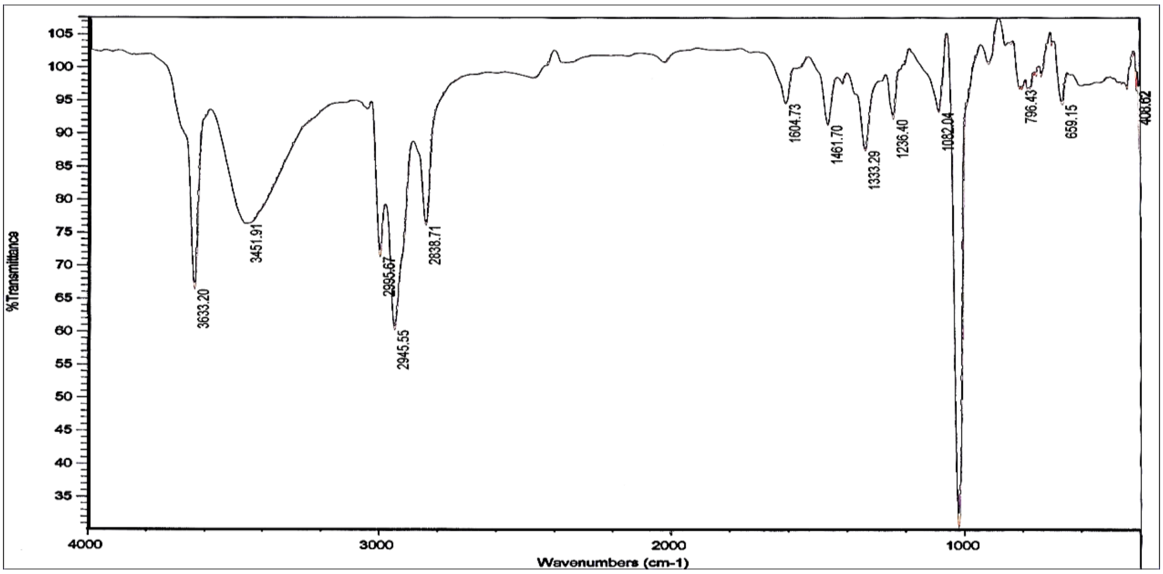

Fig. S2a. The IR spectrum of Delphitisine (**3**) in $CHCl_3$.

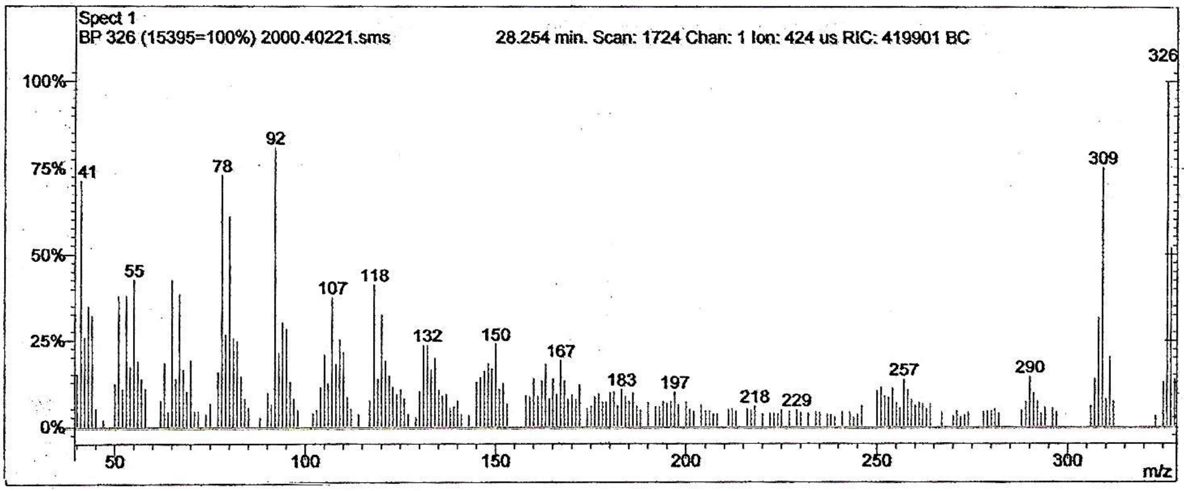

Fig. S2b. The EIMS spectrum of Delphitisine (**3**).

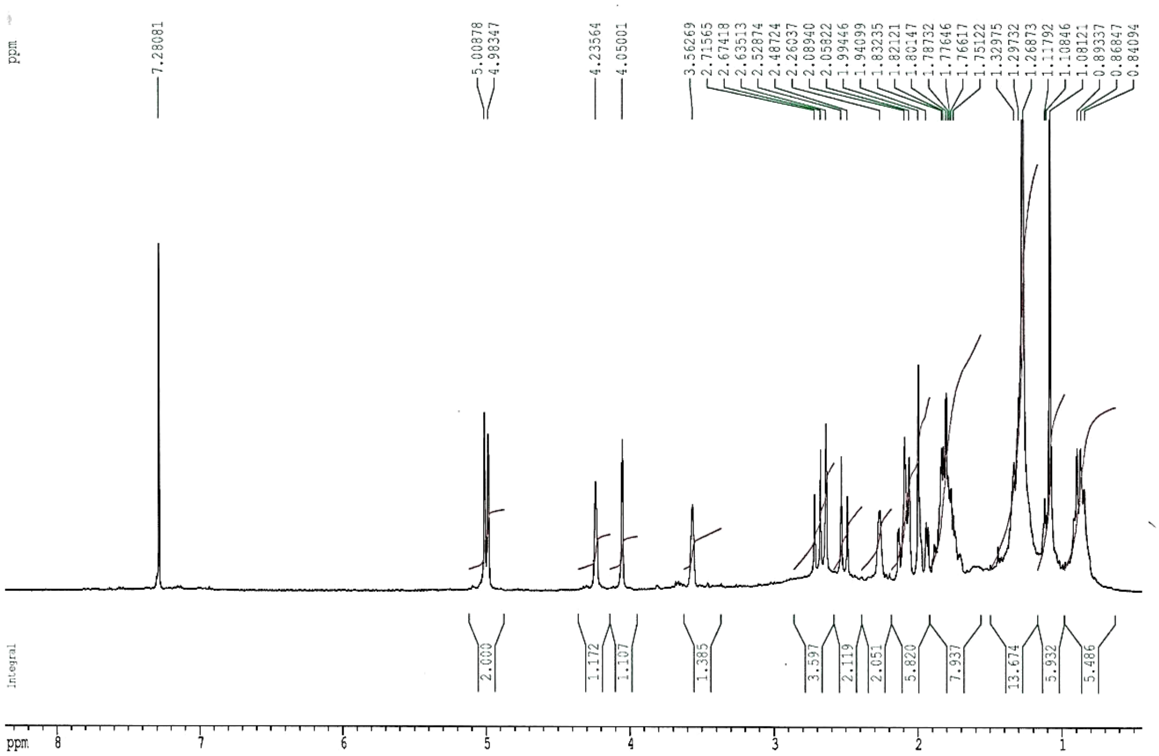

Fig. S2c. The ^{1}H-NMR spectrum of Delphitisine (**3**) in $CDCl_3$ Bruker 300 MHz.

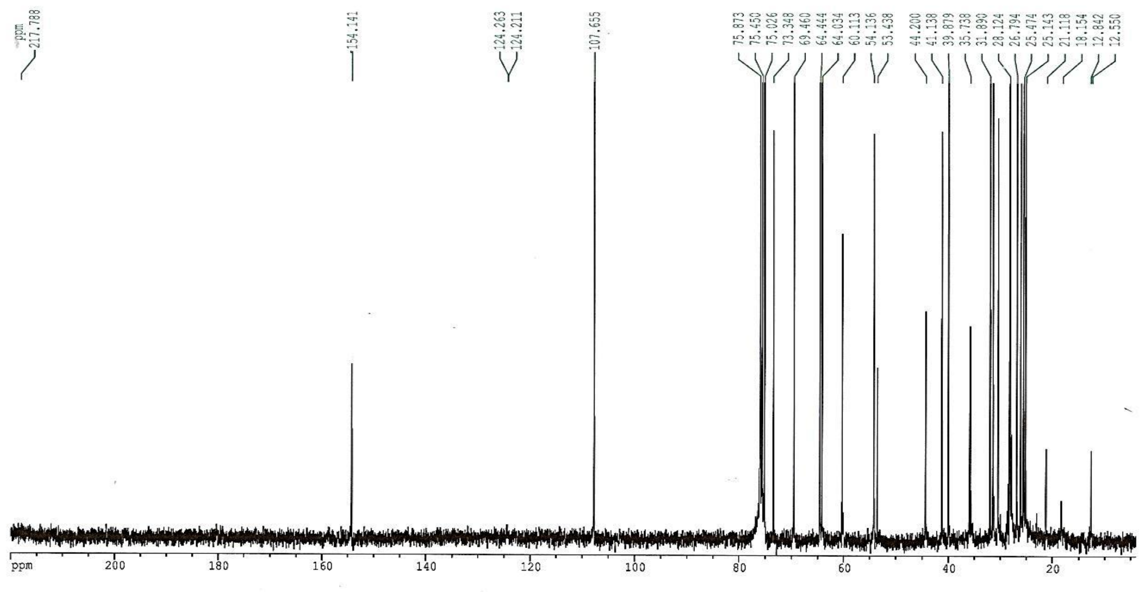

Fig. S2d. The ^{13}C-NMR spectrum of Delphitisine (**3**) in $CDCl_3$ Bruker 300 MHz.

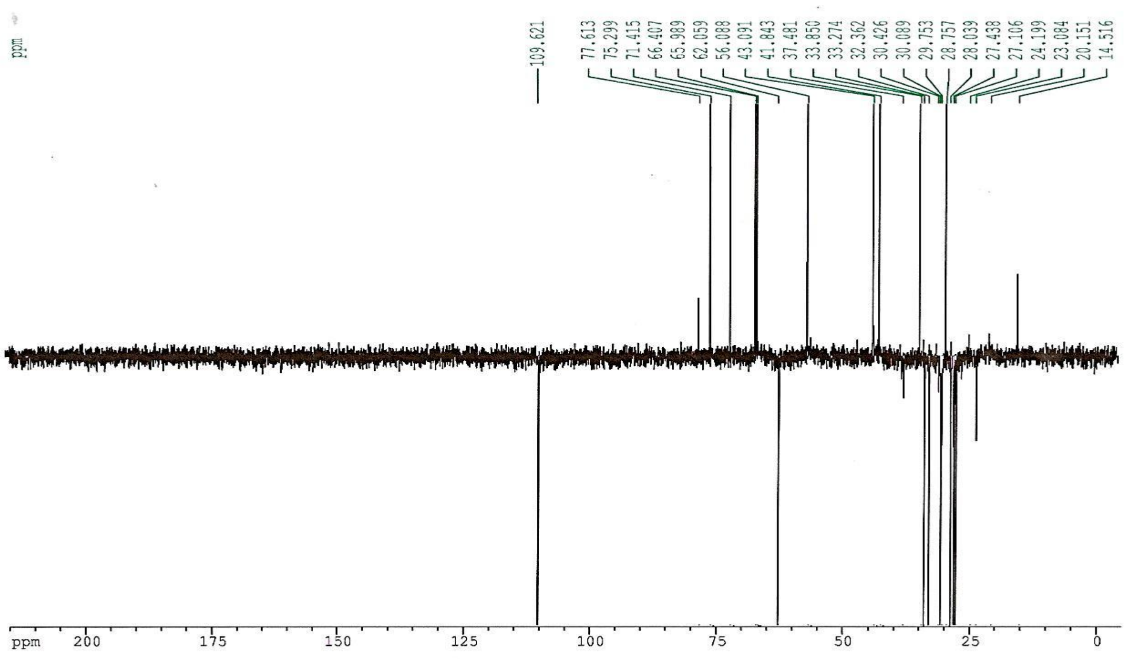

Fig. S2e. The DEPT-135 spectrum of Delphitisine (**3**) in $CDCl_3$ Bruker 300 MHz.

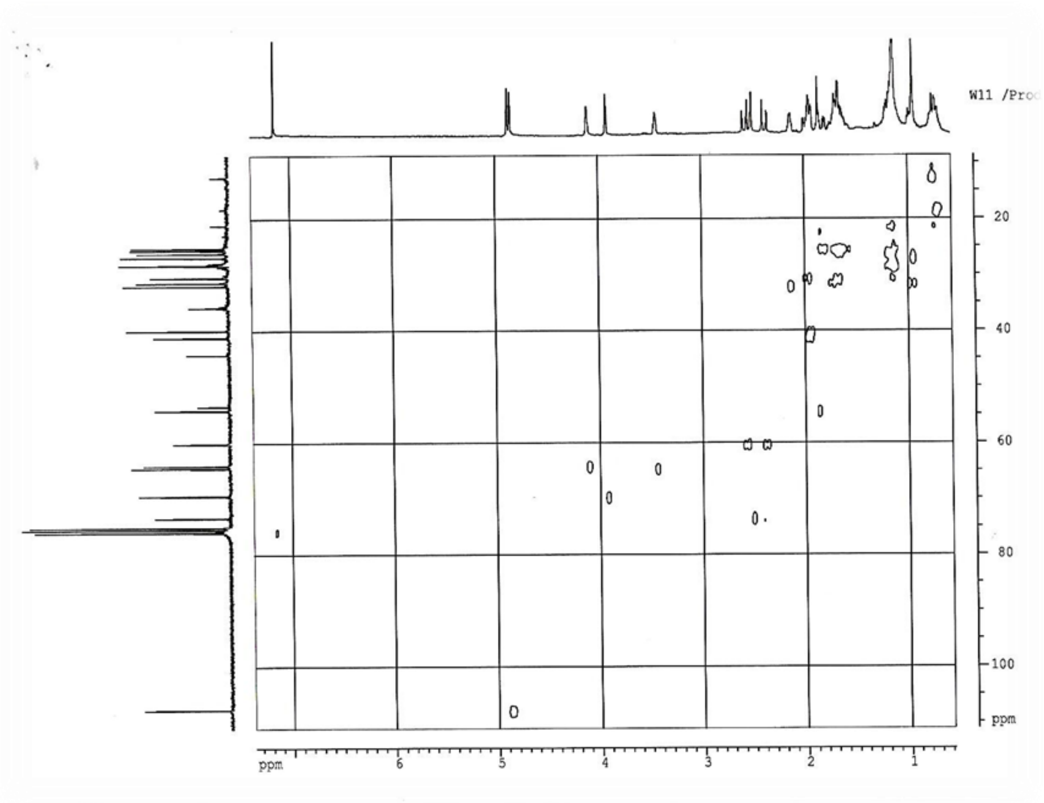

Fig. S2f. The $^{13}C/^{1}H$ HMQC spectrum of Delphitisine (**3**) in $CDCl_3$ Bruker 300 MHz.

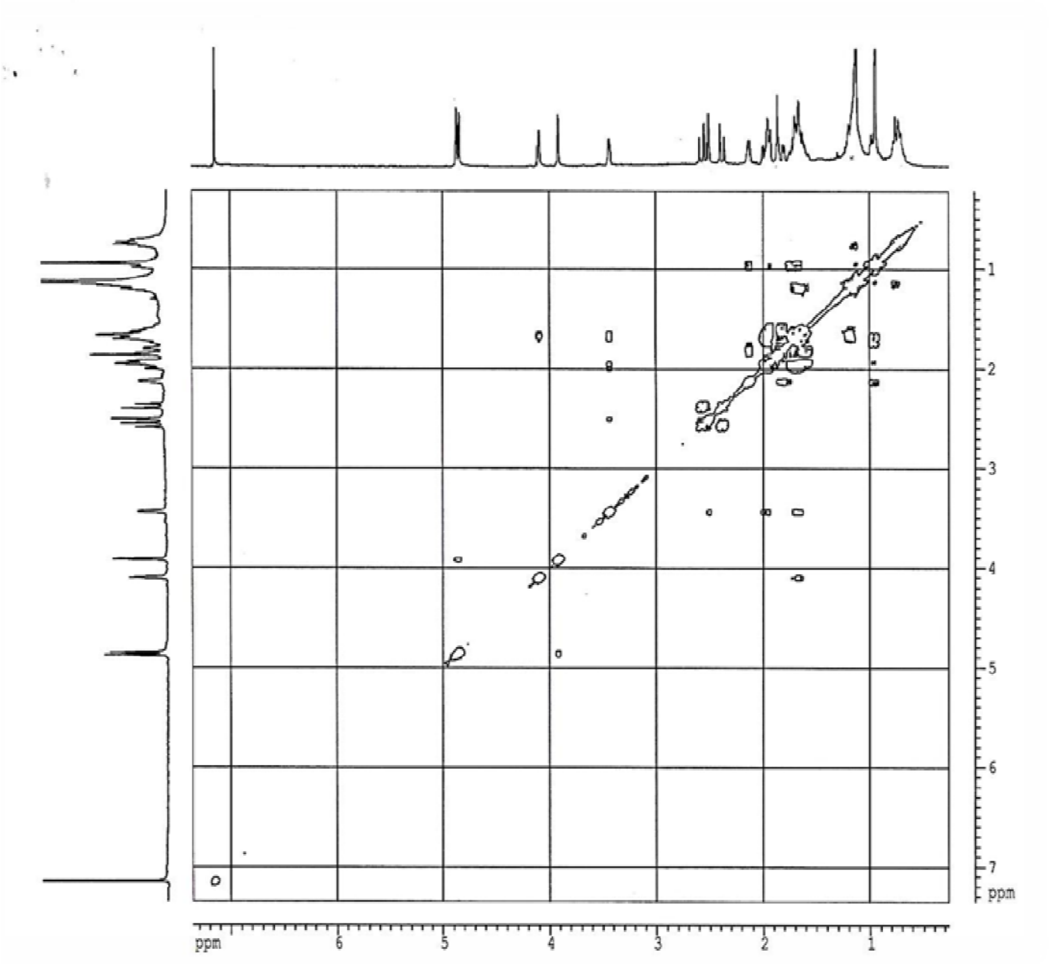

Fig. S2g. The ^{1}H/^{1}H DQF-COSY spectrum of Delphitisine (**3**) in $CDCl_3$ Bruker 300 MHz.

Fig. S2a-g. Spectral characterization of Delphitisine (**3**).

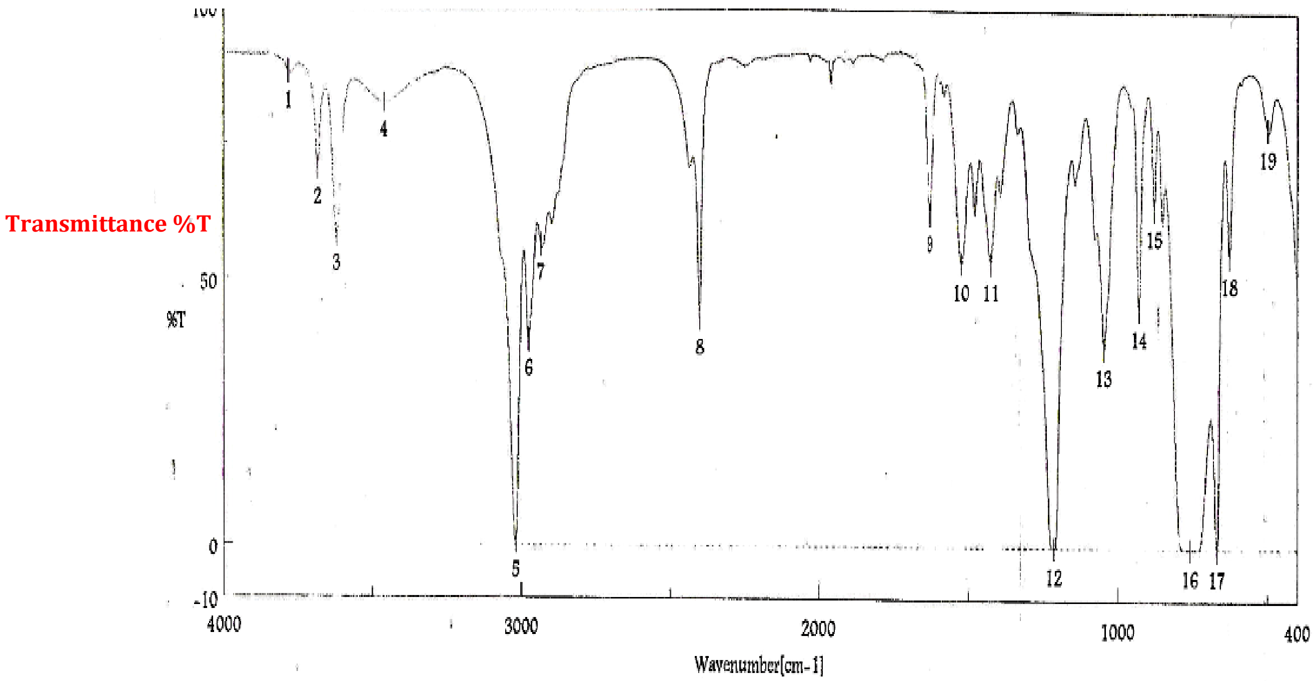

Fig. S3a. The IR spectrum of Hydrodavisine (4) in MeOH.

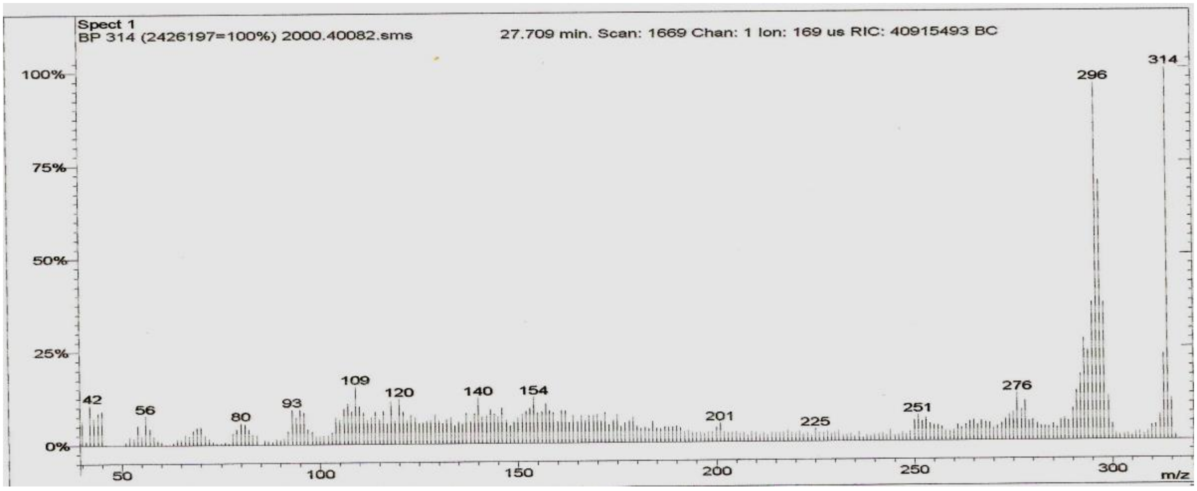

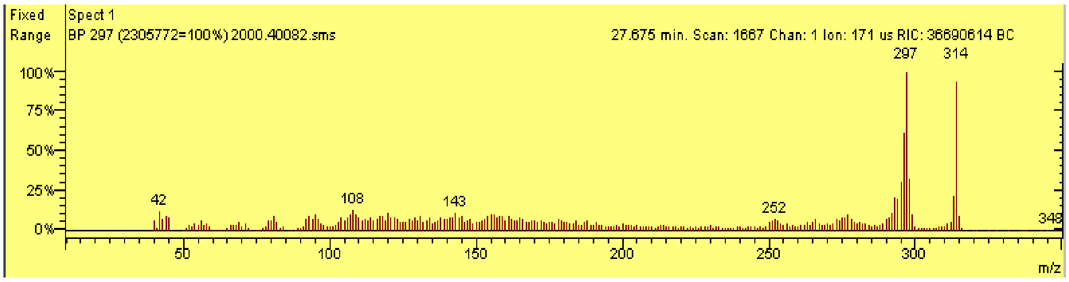

Fig. S3b. The EIMS spectrum of Hydrodavisine (4).

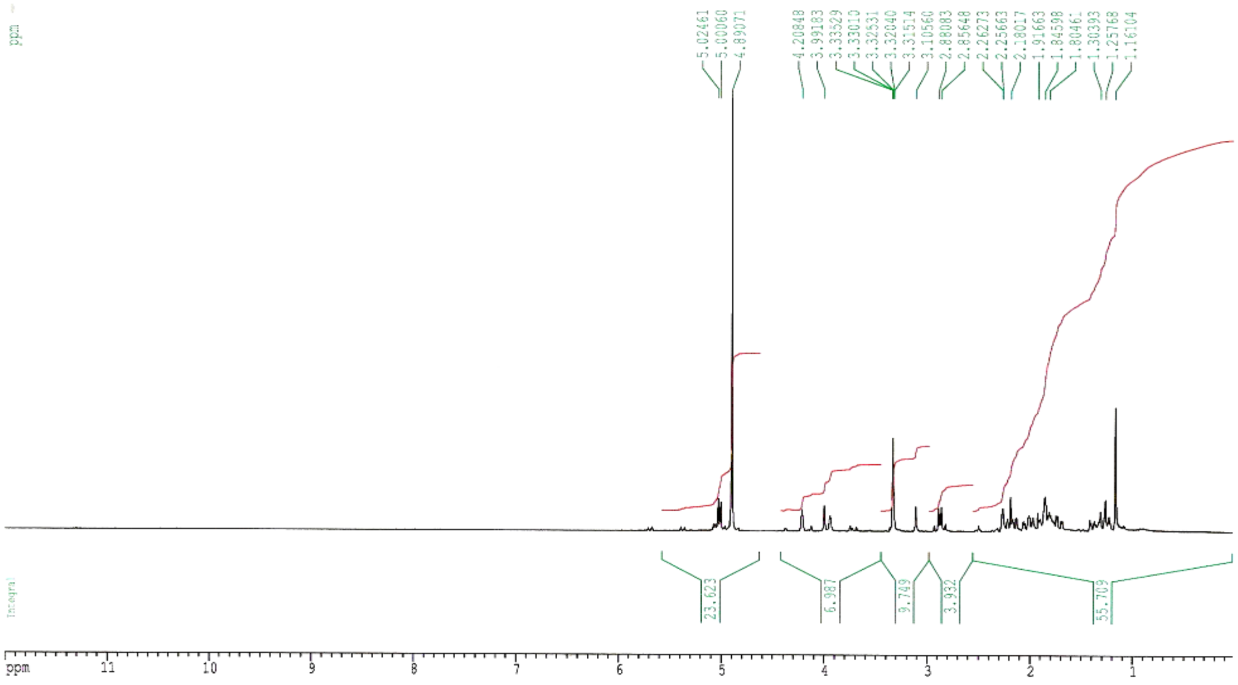

Fig. S3c. The ^{1}H-NMR spectrum of Hydrodavisine (4) in CD_3OD Bruker 300 MHz.

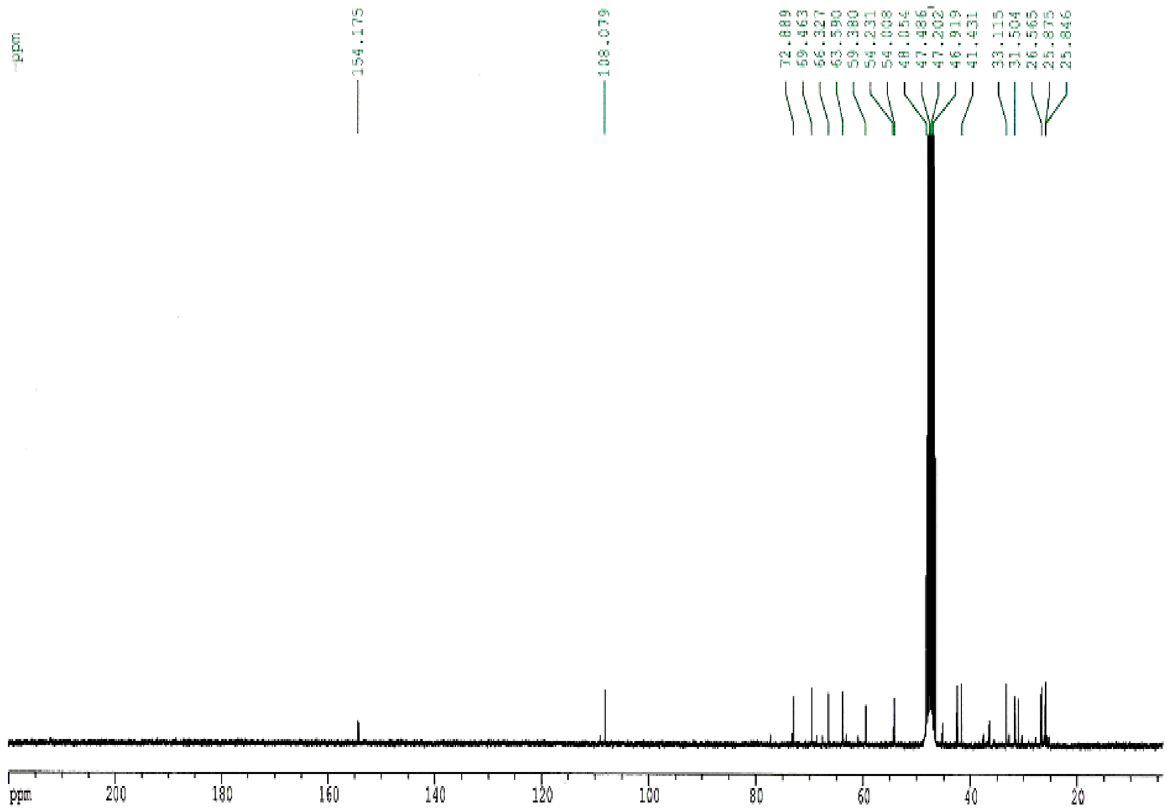

Fig. S3d. The ^{13}C-NMR spectrum of Hydrodavisine (4) in CD_3OD Bruker 300 MHz.

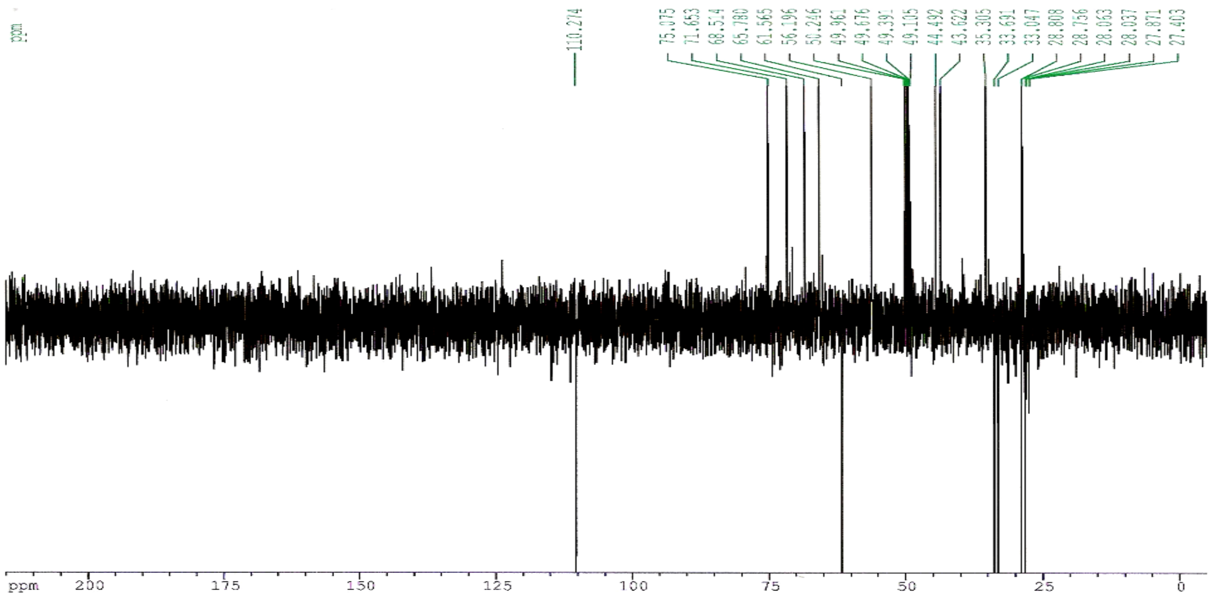

Fig. S3e. The DEPT-135 spectrum of Hydrodavisine (4) in CD_3OD Bruker 300 MHz.

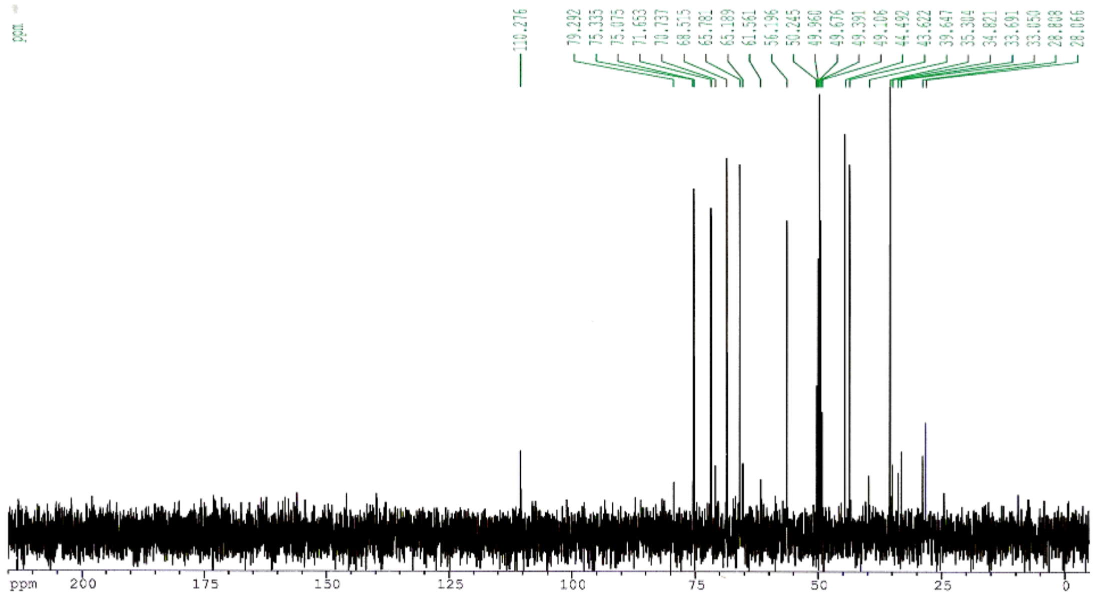

Fig. S3f. The DEPT-90 spectrum of Hydrodavisine (4) in CD_3OD Bruker 300 MHz.

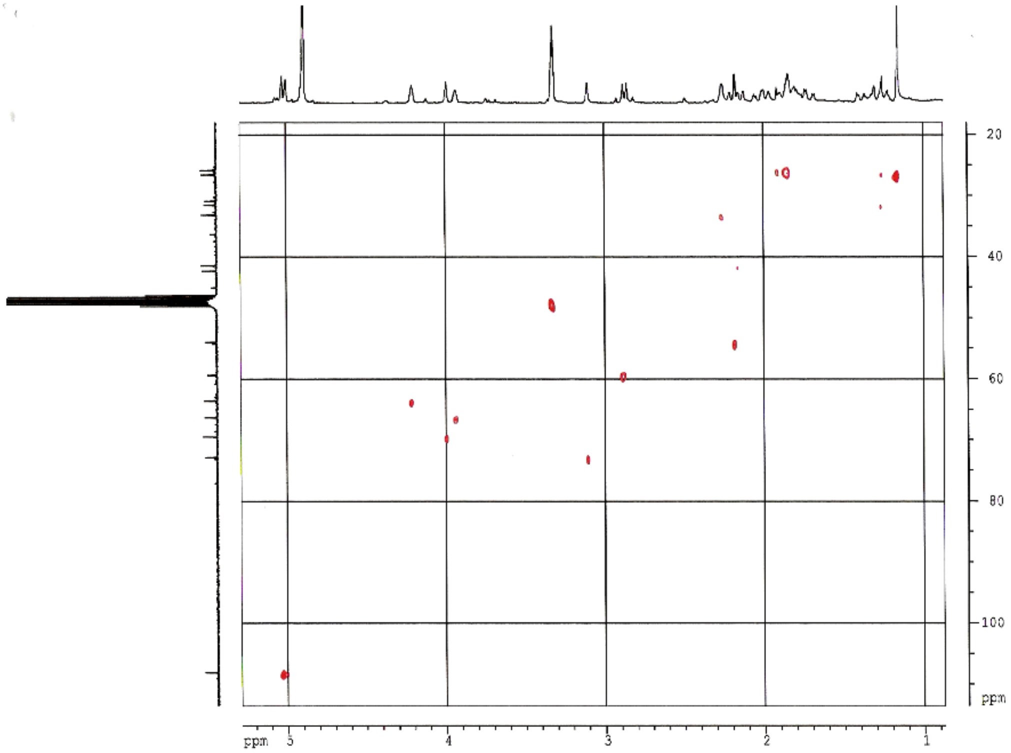

Fig. S3g. The $^{13}C/^{1}H$ HMQC spectrum of Hydrodavisine (4) in CD_3OD Bruker 300 MHz.

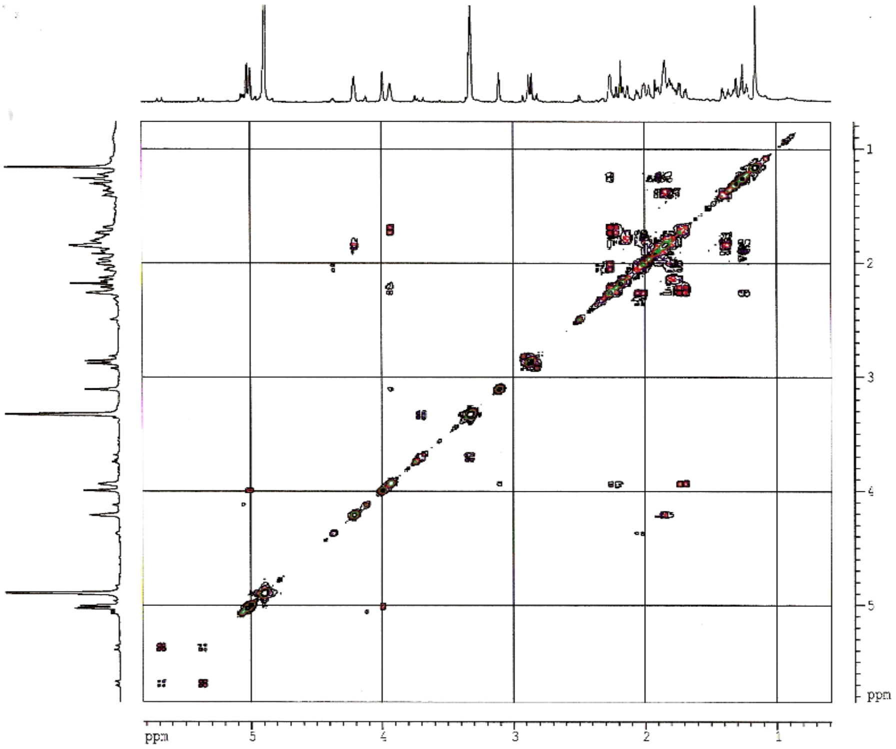

Fig. S3h. The $^{1}H/^{1}H$ COSY spectrum of Hydrodavisine (4) in CD_3OD Bruker 300 MHz.

Fig. S3a-h. Spectral characterization of Hydrodavisine (4)

Table S1. ^{1}H, ^{1}H COSY (Correlation Spectroscopy Homonuclear) and HMQC(Heteronuclear Multiple Quantum Coherence) NMR data for Delcarpum (**1**).

Proton		*Correlated atom*	
		HMQC	*COSY*
1	3.22 *m*	82.3 *d*	H-2α, H-2β, H-12α (*vw*)
2α	1.87 *m*	24.6 *t*	H-2β, H-3, H-1
2β	2.15 -		H-2α, H-3, H-1
3	1.25 *m*	.288 *t*	H-2*b*, H-2α
5	1.50 *s*	54.0 *d*	H-17(*m*), H-7, H-6α
6α	5.29 *d* (7.53)	71.4 *d*	H-7, H-5
7	3.17 -	35.2 *d*	H-6α, H-5
9	3.35 -	.406 *d*	H-14, H-10
10	2.15 -	44.3 *d*	H-9, H-12α, H-12β (*w*)
12α	1.97 -	28.2 *t*	H-12β, H-13, H-10, H-1(*vw*)
12β	2.50 *dd* (3.17, 12.6)		H-12α, H-10 (*w*)
13	2.32 *t* (5.54)	36.4 *d*	H-12*a*, H-14
14β	4.67 *t* (4.71)	75.1*d*	H-13, H-9, H-16α (*vw*)
15β	4.18 *d* (6.91)	74.1 *d*	H-16a
16α	3.11 -	91.4 *d*	H-15α, H-14 (*vw*), H-16' (*v.w*)
17	3.22 -	62.8 *d*	H-5
18	0.85 *s*	25.2 *q*	H-3, H-6''
19α	2.08 *d* (5.78)	56.8 *t*	H-19β
19β	2.86 *d* (11.93)		H-19α
20α	2.63 *m*	48.5 *t*	H-20β, H-21
20β	2.72 *m*		H-20α, H-21
21	1.14 **t** **(6.99)**	11.9 *q*	H-20α, H-20β
14''	1.97 -	20.2 *q*	-
6''	1.97 -	20.6 *q*	H-18
8'	3.17 -	48.6 *q*	-
1'	3.32 *s*	55.0 *q*	-
16'	3.45 *s*	56.2 *q*	H-16α (*v.w*)

Chemical shifts in δ (ppm) down-field from TMS. Coupling constants in parentheses in Hz.

Table S2. ^{1}H, ^{1}HCOSY(Correlation Spectroscopy Homonuclear) and HMQC(Heteronuclear Multiple Quantum Coherence) NMR data for Peregrine (**2**).

**Proton**		**Correlated atom**	
		**HMQC**	**COSY**
1	3.11-	84.1	H-2α , H-2β, H-1'
2α	2.01 -	25.9	H-2β H-1
2β	2.08 -		H-2α H-1
3α	1.25 -	.365	H-3β
3β	1.57 -		H-3α
5	1.48 s	55.7	H-17
6	5.25 *d* (7.28)	72.8	H-7
7	2.75 *d* (7.29)	41.7	H-6
9	3.05 -	44.0	H-14, H-10
10	2.01 -	45.6	H-9
12α	1.88 *m*	28.0	H-12β, H-13 , H-10 H-17(v.w)
12β	2.25 *dd* (5.31)		H-12α, H-10(w)
13	2.35 t (5.67)	37.9	H-12α (w) H-12β(m), H-14
14	4.02 *q* (10.59)	74.8	H-13, H-9, H-14' (OH)(w)
15	2.08 -	32.5	H-16
16	3.39 -	81.9	H-15
17	3.16 *d* (2.07)	64.3	H-5(m), H-1' (v.w)
18	0.85 s	25.3	-
19α	2.08 -	.569	H-19β
19β	2.61 *d* (11.90)		H-19α
20	2.47 m	48.7	H-21
21	1.07 *t* (7.12)	13.1	H-20
1'	3.28 *s*	55.6	H-1(v.w) H-17(v.w)
6''	2.08 -	21.2	-
8'	3.11 -	47.7	-
16'	3.39 -	.559	-
14' (OH)	3.70 *d* (6.48)	-	-

Chemical shifts in δ (ppm) down-field from TMS. Coupling constants in parentheses in Hz.

Table S3. **^1H, ^1HCOSY***(Correlation Spectroscopy Homonuclear) and HMQC(Heteronuclear Multiple Quantum Coherence)* **NMR data. for Delphitisine (3).**

Proton		Correlated atom	
		**HMQC**	**COSY**
1α	2.67 *s*	73.3	H-20
2α	1.79 *m*	25.1	H-2β H-3
2β	1.94 *m*		H-2 α
3	1.29 *m*	28.1	H-2 α, H-18(*w*)
4	-	35.7	-
5	1.99 *m*	54.1	H-7β (*w*)
6	4.23 *m*	64.2	H-7α
7α	1.29 *m*	26.0	H-7b , H-6
7β	1.79 *m*		H-7α
8	-	44.1	-
9	2.26 *d* (2.29)	31.8	H-11α, H-13β
10	-	53.4	-
11α	1.10 *m*	31.3	H-11β, H-12(*w*)
11β	1.79 *m*		H-11α , H-12
12	2.07 *m*	39.8	H-11β, H-11α (*w*)
13α	1.79 *m*	25.4	H-13β , H-14
13β	1.94 *m*		H-13α , H-9
14	2.07 *m*	41.1	H-13α
15α	4.05 *s*	69.4	H-17
16	-	154.1	-
17	4.99 *d* (7.60)	107.6	H-15α
18	1.08 *s*	26.7	H-3(*w*)
19α	2.50 *d* (12.45)	60.1	H-19β
19β	2.69 *d* (12.44)		H-19α
20	3.56 *m*	64.4	H-1, H-21α , H-21β
21α	1.79 *m*	30.4	H-21β, H-20
21β	2.07 *m*		H-21α, H-20
1'-OH	1.77 *m*	-	
15'-OH	1.77 *m*	-	

Chemical shifts in δ (ppm) down-field from TMS. Coupling constants in parentheses in Hz.

Table S4. ^{1}H, ^{1}HCOSY *(Correlation Spectroscopy Homonuclear) and HMQC(Heteronuclear Multiple Quantum Coherence)*NMR data for ***Hydrodavisine 4.***

Proton		Correlated atom	
		**HMQC**	**COSY**
1α	3.10 *s*	72.9	H-20
2	1.38 *m*	31.5	H-3α , H3β
3α	1.84 *m*	25.9	H-3β ,H-2
3β	1.90 *m*		H-3α
4	-	35.4	-
5	2.21 *m*	54.0	H-20, H-11
6	4.20 *s*	63.6	H-7
7	1.84 *m*	25.9	H-6, H-9
8	-	36.3	-
9	2.25 *m*	33.1	H-14, H-20, H-7, H-13β
10	-	54.2	-
11	1.70 *m*	30.9	H-5, H-13β, H-20
12	2.11 *m*	42.3	H-9, H-13β
13α	1.38 *m*	26.6	H-13β, H-9
13β	1.84 *m*		H-13α, H-12, H-11, H-9, H-14
14	2.11 *m*	41.4	H-9, H-13β
15α	3.99 *s*	69.5	H-17
16	-	154.2	-
17	5.01 *d* (7.21)	108.1	H-15α
18	1.16 *s*	26.6	-
19	2. 86 *q* (12.29)	59.4	-
20	3.93 *s*	66.3	H-1α, H9, H-5, H-11
-$^{+}$NH	3.74 *m* ($^{+}$NH)	-	-
1'-OH	4.89 *br. s*	-	-
15'-OH	4.89 *br. s*	-	-
2H_2O	4.89 *br. s*	-	-

Chemical shifts in δ (ppm) down-field from TMS. Coupling constants in parentheses in Hz.
